# When Covid-19 first struck: Analysis of the influence of structural characteristics of countries - technocracy is strengthened by open democracy

**DOI:** 10.1371/journal.pone.0257757

**Published:** 2021-10-04

**Authors:** Michael J. Rigby, Kinga Zdunek, Fabrizio Pecoraro, Marco Cellini, Daniela Luzi

**Affiliations:** 1 School of Social, Political and Global Studies and School of Primary, Community and Social Care, Keele University, Keele, United Kingdom; 2 Public Health Department, Medical University of Lublin, Lublin, Poland; 3 Institute for Research on Population and Social Policies, National Research Council (IRPPS-CNR), Rome, Italy; University of Lincoln, UNITED KINGDOM

## Abstract

**Context:**

The Covid-19 pandemic hit the developed world differentially due to accidental factors, and countries had to respond rapidly within existing resources, structures, and processes to manage totally new health challenges. This study aimed to identify which pre-existing structural factors facilitated better outcomes despite different starting points, as understanding of the relative impact of structural aspects should facilitate achieving optimal forward progress.

**Methods:**

Desk study, based on selecting and collecting a range of measures for 48 representative characteristics of 42 countries’ demography, society, health system, and policy-making profiles, matched to three pandemic time points. Different analytic approaches were employed including correlation, multiple regression, and cluster analysis in order to seek triangulation.

**Findings:**

Population structure (except country size), and volume and nature of health resources, had only minor links to Covid impact. Depth of social inequality, poverty, population age structure, and strength of preventive health measures unexpectedly had no moderating effect. Strongest measured influences were population current enrolment in tertiary education, and country leaders’ strength of seeking scientific evidence. The representativeness, and by interpretation the empathy, of government leadership also had positive effects.

**Conclusion:**

Strength of therapeutic health system, and indeed of preventive health services, surprisingly had little correlation with impact of the pandemic in the first nine months measured in death- or case-rates. However, specific political system features, including proportional representation electoral systems, and absence of a strong single party majority, were consistent features of the most successful national responses, as was being of a small or moderate population size, and with tertiary education facilitated. It can be interpreted that the way a country was lead, and whether leadership sought evidence and shared the reasoning behind resultant policies, had notable effects. This has significant implications within health system development and in promoting the population’s health.

## Introduction

The Covid-19 pandemic exploding on the world from early 2020 gave a new and severe challenge to nations’ health systems. Urgent and radical decisions had to be made by policy makers, implemented by the staff and facilities of the health system, and responded to by society. It soon became clear that the outcome of these responses, in the first months of 2020, had significantly different degrees of outcome success.

While much critical narrative is being written about the pandemic from public health practice and health policy viewpoints, little has been reported about the effects of different health resource structures of countries when first hit by the pandemic, and which characteristics were associated with successful control in the early months. Though most unwelcome in its nature, the pandemic can be considered as providing a real-world stress-testing of health systems’ public health active response and emergency reconfiguration capacities, and a unique opportunity to seek to identify the most beneficial characteristics of health systems, populations, and health policy making.

While this study sought to address the measurement of likely relevant structural factors *ab initio*, it is important to consider as background prior World Health Organisation guidance on preparedness for health emergencies [1]. Six aspects are put forward: leadership and governance in order to assure the crisis management programme; health workforce adequate to ensure an appropriate level of responsiveness; equitable access to medical products, vaccines and technologies; well-functioning health information system to provide efficient early-warning systems and the overall management of information; good health financing system providing appropriate level of financial protection as well as ensuring provision of essential services; and service delivery which guarantees continuity of service provision during a crisis. Further, it might be hypothesised that countries which had most resources and health system spend would be the most powerful and nimble to respond effectively to a new health threat at scale, and that those with effective preventive health programmes and salutogenic public behaviour would be most likely to elicit the strongest practical population responses and preventive behaviour compliance, and would therefore be best placed to respond effectively to the first impact of the virus.

This study sought to examine structural foundations and guiding forces by comparing measures of pre-Covid characteristics in a range of countries, mapped to early outcome measures, to identify the most effective response and resilience facilitators. It was acknowledged that physical resources would be more likely to have objective pre-existing measures, with objectively obtained comparable attitudinal measures being more challenging to identify.

### Purpose and objectives

The aim was to identify which health system, preventive health and societal characteristics had most influence on the response in early months to the Covid-19 pandemic. Framed by the analysis of Donabedian, these are items of Structure [2]–the quasi-fixed health and population assets present which form the foundation for the means of response when the unexpected pandemic struck. This should enable better understanding of managing whole society health challenges, and assist with building optimal health system support in the post-Covid era. The purpose was not to evaluate individual process responses, which rightly many are doing [3–5], but to assess the influence of pre-existing underlying structures and systems in giving initial resilience.

## Methods

### Study countries

The aim was to study a range of countries which were broadly comparable in a development context, but which had notable differences regarding the types of health system and societal attitudes. A global wide range would constitute a virtual laboratory.

Countries in the European Union (EU), other countries in Europe with similar levels of development, North American countries, and countries elsewhere in the Organisation for Economic Cooperation and Development (OECD) met these criteria, with the added advantage that many comparable data sets and definitions were available. Most global regions were covered. The final selection was the 27 countries in the EU, the contiguous countries of Iceland, Norway, Switzerland, and the United Kingdom, together with the remaining OECD countries—a total of 42. This in no way diminishes the importance of an effective Covid-19 response for the populations of countries elsewhere, particularly in the lower and middle income countries (LMIC), but the context, factors, and available indicators for these countries are very different. Though a number of countries in South East Asia have had very positive results in managing the Covid-19 pandemic, they had the advantage of exposure to the prior SARS pandemic which would have had a training effect for health systems and populations [6]. The BRICS countries (Brazil, Russia, India, China, and South Africa) are also globally significant, but each is sufficiently different, vast, and with its own internal structures and power levers, that lessons from these are not necessarily transferable.

### Time points and Covid measures

To achieve the study’s objective of assessing the influence of different Structural assets as they existed in the steady state before the pandemic, consideration was restricted to the period before semi-fixed components could change, or population behaviours be modified by experience and growing realisation of the pandemic’s deep effects. Three broad initial phases were hypothesised, with the study focussed on the initial two:

- **Bewildering Arrival**–the novel disease is suddenly present, with bewilderingly different impacts in some countries [7], not least because of the initially not understood asymptomatic infectious stage [8], with authorities wondering how to respond. This was taken to last up to late March 2020.
- **Emergency Systematic Responses**–from Spring 2020 the spread and potentially disastrous effects of the pandemic were clear, and countries sought to organise system-wide society-wide responses, but perforce based on current resources and attitudes. This period was taken to run from April to November 2020.
- **Initial Steady State**–By late Autumn 2020 most countries had established radically new patterns of public health pandemic treatment, and other actions balancing health, economic, and education needs. This study stopped at this point, as we were studying influences on the initial emergency responses.

The first challenge was finding comparable measures of pandemic impact in each country. Death is the ultimate outcome state, but excludes the many who contract the disease then recover, either completely or with long-term sequelae [9]. Cases of infection is theoretically the optimum measure, but in the early bewildering arrival stage testing and tracing systems were very imperfect, with very different (and generally low) proportions of cases being detected and reported, and so early case data cannot be compared between countries. This variability of case assessment continued in many countries into the Emergency System Response stage. Deaths are reasonably reliably measured and attributed in all the study countries; some differences in detail of attribution are insufficient to skew major differences in death rates.

This study therefore took three outcome measures:

- Total deaths attributed to Covid-19 per million population by 31 May 2020, as an indicator of outcome of system response by late-March, allowing for a fatal outcome time lag, thus giving a measure of the Bewildering Arrival phase specific to each country.
- Total deaths attributed to Covid-19 per million population by 30 November 2020, as an indicator of outcome of immediate system response in the subsequent Emergency System Response six months
- Cases per 100,000 population, 14 day moving average, early December, necessarily assuming that case finding and recording systems were reliable by this time.

### Analyses performed

First, simple correlation was performed to relate numerate structural variables to the pandemic impact at the stated time points. In particular, Pearson’s pairwise correlation was applied, and for each correlation the level of significance was calculated.

Second, a cluster analysis ascertained whether groups of countries could be identified, and then related these to the variables. Based on deaths per million inhabitants at the end of May and the increase over the next six months, we adopted a hierarchical clustering approach that creates a complete data partition applying the Ward method [10] based on the Euclidean distance/proximity matrix. Moreover, to assess how the identified clusters relate with the variables selected, we calculated the average value of each variable for each cluster.

Finally, an econometric model was developed to assess the strength of selected structural variables’ influence on countries’ outcomes. To estimate the effect of the variables selected on our dependent variables, we employed an Ordinary Least Squares (OLS) linear regression estimation. Since preliminary tests showed that most of our independent variables did not meet the OLS linearity assumption, we transformed all the variables into natural logs. Log-log regressions, furthermore, are often preferred since they allow for a straightforward interpretation of the coefficients. The goodness of our model was assessed through several post-estimation tests that provided positive results. In particular, the Breusch-Pagan [11] and Cook-Weisberg [12] tests confirmed the variance of the residuals to be constant, excluding the presence of heteroscedasticity. The variance inflation factors test showed the absence of multicollinearity. Finally, the link test and Ramsey regression specification-error test for omitted variables confirmed the goodness of the model’s specification.

## Findings

### Country demographic, socio-economic, societal, health, and political characteristics

After consideration of aspects of health structure and resources, society, and decision-making, coupled with detailed review of data options, analysis was based on six themes containing a total of 48 data items:

- Demographic and Socio-economic indicators (12 measures)
- Societal Values (11 measures)
- Public Trust and Awareness (6 measures)
- Public Health (6 measures)
- Healthcare System (6 measures)
- National Political Process (7 measures).

Topic selection was based on a considered rationale, moderated by the need for data availability in comparable form, with the reasons for selection of each item given in S1 Table. Some aspects of the WHO risk response framework [4] Were considered to be Process measures, more related to focussed response to a crisis and also less easy to be subjected to objective baseline measurement, and could not be assessed. All measures selected for the study were numeric ranges, except for three Political Process characteristics.

The full 48 item by 42 countries matrix of values captured and used is presented in S2 Table, and the data sources and accessed dates in S3 Table. Almost all numeric items were measured within the last three years. For 25 (52.1%) of the measures complete data sets were obtained. For five of the Public Trust and Awareness measures there were no comparable studies in the rest of the world countries. Some individual items were not available for some small countries. Full data sets were obtained for eleven of the thirteen Demographic and Socio-economic measures and for five of the seven Political System measures. S4 Table gives the number of national values captured for each Measure. All data could be considered static in the short term except age of prime minister–three countries changed prime minister in the study period and the date-relevant age was used in calculations. Data definitions and sources are given in S3 Table, while Prime Minister details are in S5 Table.

### Health impact of Covid-19 pandemic first waves

The Covid-19 total mortality data per million, cumulative at 31 May and 30 November 2020, and the Case rate by 14-day average as at 6 December, were obtained. Table 1 shows these data.

**Table 1:** Covid-19 incidence in European union and OECD countries at three time points in 2020.

	Deaths per million	Deaths per million	14 Day Case Rate per 100,000
	31 May 2020	30 Nov 2020	6 Dec 2020
Austria	74.17	353.5	171.5
Belgium	807.10	1436.2	272.9
Bulgaria	20.15	580.7	573.5
Croatia	25.09	435.1	1154.1
Cyprus	19.41	55.9	425.3
Czechia	29.79	777.6	501.7
Denmark	98.58	144.5	331.1
Estonia	47.49	89.0	386.8
Finland	57.03	72.0	108.8
France	440.78	809.2	230.4
Germany	101.45	199.3	304.8
Greece	16.79	230.8	228.0
Hungary	54.24	499.3	774.2
Ireland	334.16	415.8	77.6
Italy	551.42	919.2	545.8
Latvia	12.72	109.2	418.9
Lithuania	25.71	185.9	1070.4
Luxembourg	175.73	512.8	1181.8
Malta	20.38	310.3	305.9
Netherlands	347.30	551.7	411.4
Poland	28.03	453.2	555.1
Portugal	136.91	441.8	609.8
Romania	65.13	589.0	492.1
Slovakia	5.13	153.7	370.7
Slovenia	51.95	690.3	984.7
Spain	560.20	963.9	272.5
Sweden	454.79	661.5	713.8
Iceland	29.30	76.2	58.0
Norway	43.53	61.2	111.4
Switzerland	191.34	556.4	627.5
United Kingdom	550.70	862.4	319.0
Australia	4.00	35.6	0.6
Canada	187.40	321.8	220.5
Chile	52.15	806.1	102.1
Colombia	17.49	722.6	239.3
Israel	32.81	331.8	181.0
Japan	7.04	16.4	23.7
Korea S.	5.27	10.3	13.3
Mexico	75.85	821.7	105.3
New Zealand	4.56	5.2	1.0
Turkey	53.53	163.0	112.7
United States	313.54	810.1	753.5

### The correlations of structural characteristics with outcomes

As the first analysis, to identify which national characteristics or resource patterns had most effect (positive or negative) on the outcomes of the pandemic in the first months, the 45 numeric measures were matched to each country’s Covid pandemic situation at the three time points, as paired dependent and independent variables, for three groupings of the study countries, and the 405 correlation calculations are shown in Table 2. In that table, weak correlations between 0.40 and 0.59 are highlighted by a dotted cell margin; stronger correlations from 0.60 upwards by a solid box; perverse direction correlations where the measure correlates with a worse disease situation are shaded.

**Table 2:** Correlations between selected structural measures and mortality and incidence data.

**2a. Demographic and Socio-economic**																																							
			Popn (mill)			Dependent Popn			Urban Pop. %			Urban Pop (mill)		Pop’n. Den’y			Pop’n 65+ %		GDP per capita				Gini Index				IncomShare low 10%			Mat’al Pov’ty				Tert. Educ. Enrol				Tert. Educ. Comp.	
			2019			2019			~2017			~2017		2018			2019		2019				2017							2019				2018				2010	
**Deaths to 31 May 2020**																																							
EU 27			0.40*			0.24			0.47*			0.44*		0.05			0.02		0.35				0.00				-0.08			-0.06				0.04				0.13	
EU++ 31			0.49**			0.27			0.41*			0.53**		0.09			0.07		0.25				0.10				-0.12			-0.08				-0.01				0.12	
OECD 42			0.20			0.18			0.25			0.20		0.06			0.16		0.28				-0.06				0.02			-0.14				-0.05				0.07	
**Deaths to 30 November 2020**																																							
EU 27			0.32			0.16			0.26			0.33		0.07			0.11		0.07				0.05				-0.01			-0.07				-0.13				-0.15	
EU++ 31			0.40*			0.19			0.17			0.41*		0.12			0.20		-0.03				0.09				-0.09			-0.09				-0.17				-0.13	
OECD 42			0.25			0.02			0.06			0.24		0.02			-0.04		-0.08				0.17				-0.05			0.06				-0.28				-0.19	
**Cases 14 day average to 6 December 2020**																																							
EU 27			-0.25			-0.25			-0.21			-0.27		-0.13			-0.05		-0.04				0.13				-0.09			0.17				-0.47*				-0.12	
EU++ 31			-0.20			-0.22			-0.29			-0.22		-0.08			0.08		-0.12				0.20				-0.17			0.14				-0.45*				-0.13	
OECD 42			-0.04			-0.08			-0.39*			-0.05		-0.07			0.25		0.01				-0.07				0.06			-0.05				-0.41*				-0.11	
**2b. Societal Values**																																							
		Human Dev. Index			World Happ’s Index			OECD Life Satisf.			Trust news media				Trust Writtn Press			Int’net Users			Civil Society Partp’n				Public Service Fragilit				Good/ v good health				Relig’n Imp’tn				Relig’n Wee’ly		
		2017			2020			2017			2020				2019			2017			2017				2017				2017				2018				2018		
**Deaths to 31 May 2020**																																							
EU 27		0.47*			0.32			0.25			0.00				0.29			0.29			0.27				-0.29				0.35				-0.27				-0.10		
EU++ 31		0.38*			0.26			0.13			-0.10				0.09			0.24			0.19				-0.25				0.31				-0.29				-0.12		
OECD 42		0.32*			0.27			0.14			-0.05							0.23			0.28				-0.25				0.17				-0.22				-0.13		
**Deaths to 30 November 2020**																																							
EU 27		0.12			0.04			0.02			-0.23				0.03			-0.10			-0.08				0.00				0.06				-0.17				-0.02		
EU++ 31		0.03			-0.02			-0.09			-0.29				-0.08			-0.15			-0.16				0.05				0.01				-0.17				-0.01		
OECD 42		-0.15			0.00			-0.04			-0.23							-0.25			-0.07				0.21				-0.02				0.06				0.08		
**Cases 14 day average to 6 December 2020**																																							
EU 27		-0.36			-0.31			-0.26			-0.17				-0.11			-0.18			-0.27				0.27				-0.60**				0.17				0.05		
EU++ 31		-0.41*			-0.36*			-0.32			-0.15				-0.04			-0.30			-0.35				0.32				-0.60**				0.19				0.11		
OECD 42		-0.15			-0.18			-0.18			-0.08							-0.16			-0.03				0.01				-0.51**				0.15				0.07		
**2c Public Trust and Awareness**																		**2d Public Health**																					
	Confid Health Systm			Confidsocial media			Confid Gov’t			Follow politic on TV			Follow politic social media			Follow politics on radio				Infant Mort’y				Life Expect				Cur’nt smoke			Cerv’l. Screen				MCV1 Imms				Flu vacc’n > 65
	2017			2017			2017			2017			2017			2017				2020				2020				2012			2016				2017				2019
**Deaths to 31 May 2020**																		**Deaths to 31 May 2020**																					
EU 27	0.52*			-0.24			0.11			-0.32			-0.20			-0.36		EU 27		-0.35				0.55**				-0.25			0.11				0.05				0.63**
EU++ 31	0.45*			-0.23			0.05			-0.41			-0.29			-0.42*		EU++ 31		-0.27				0.46*				-0.18			0.13				0.00				0.64***
OECD 42							0.01											OECD 42		-0.21				0.30				-0.06			0.14				-0.09				0.40*
**Deaths to 30 November 2020**																		**Deaths to 30 November 2020**																					
EU 27	-0.02			-0.12			-0.20			-0.34			-0.62**			-0.63**		EU 27		-0.18				0.32				-0.04			-0.05				0.08				0.38
EU++ 31	-0.09			-0.09			-0.21			-0.36			-0.29**			-0.63**		EU++ 31		-0.08				0.22				0.06			-0.05				0.04				0.39
OECD 42							-0.33											OECD 42		0.09				-0.05				0.01			-0.02				-0.01				0.25
**Cases 14 day average to 6 December 2020**																		**Cases 14 day average to 6 December 2020**																					
EU 27	-0.65**			0.36			0.02			-0.16			-0.06			-0.31		EU 27		0.18				-0.28				0.00			0.00				0.09				-0.43
EU++ 31	-0.69**			0.35			0.00			-0.08			-0.29			-0.30		EU++ 31		0.24				-0.34				0.15			-0.05				0.11				-0.42*
OECD 42							-0.08											OECD 42		-0.02				-0.31*				0.23			-0.02				-0.02				-0.38*
**2e Healthcare System**																		**2f Political System**																					
	Spend per capita			Doctor /1,000			Health Empl/ 1,000			Hosp. beds /1,000			Acute beds /1000			Health R&D $$$						Trust in Government				Corruptn Perceptn						Taken Scienc Advice				Age of PM			
	2017			2018			2018			2019			2017			2018						2018				2018						2020				2020			
**Deaths to 31 May 2020**																		**Deaths to 31 May 2020**																					
EU 27	0.51**			-0.04			0.14			-0.30			-0.34			0.32		EU 27				-0.15				0.33						-0.40				-0.15			
EU++ 31	0.39*			-0.18			0.07			-0.31*			-0.28			0.43*		EU++ 31				-0.11				0.30						-0.49				-0.11			
OECD 42	0.40**			0.06			0.11			0.10			-0.25			0.16		OECD 42				0.07				0.27						-0.34				-0.18			
**Deaths to 30 November 2020**																		**Deaths to 30 November 2020**																					
EU 27	0.20			-0.18			-0.02			-0.30			-0.10			0.19		EU 27				0.07				-0.04						-0.55				0.07			
EU++ 31	0.10			-0.30			-0.12			-0.31			-0.02			0.29		EU++ 31				0.09				-0.06						-0.58*				0.09			
OECD 42	0.07			-0.10			-0.16			0.10			-0.21			0.18		OECD 42				-0.04				-0.17						-0.57**				-0.01			
**Cases 14 day average to 6 December 2020**																		**Cases 14 day average to 6 December 2020**																					
EU 27	-0.28			-0.16			-0.14			0.13			0.16			-0.25		EU 27				0.06				-0.23						-0.17				0.30			
EU++ 31	-0.29			-0.19			-0.22			0.22			0.23			-0.21		EU++ 31				0.05				-0.28						-0.12				0.28			
OECD 42	-0.01			0.13			-0.05			0.05			0.02			0.18		OECD 42				0.12				-0.13						-0.11				0.04			

Asterisks report the level of significance of the correlations as follows:

*** p<0.01,

** p<0.05,

* p<0.1.

Table 2A reports on the Demographic and Socio-economic variables. Urban population percentage and total size had a weak correlation in the early stages, matching greater transmission risk in urban settings. Unexpected was the correlation with total population size. Also unexpected was the lack of influence in inter-country comparisons of proportion of persons living in poverty, the Gini index of inequality, or proportion of elderly population, since within country poverty and deprivation are considered exacerbating factors for Covid-19 vulnerability, and the elderly are a major vulnerable group. Tertiary education enrolment was linked with lower December case rates.

Table 2B shows that Societal Value items are not notably associated with any effects. By contrast, in Table 2C measures of Public Trust from the European Values Study and the World Bank show interesting correlations—trust in the health care system is intriguingly weakly linked with higher deaths at the beginning of the pandemic, has no linkage with deaths after another six months, but is more strongly linked with lower case rates by the end of the second wave. The following of politics on radio, and within the EU on social media, is correlated with lower death rates cumulative to November, but not with the other impact measures. Participation in civil society has a weak correlation within the broader European group. Confidence in government, and valuing public services, have little impact.

Table 2D shows analysis of the correlation with public health activities such as child immunisation and cervical screening, the hypothesis being that countries with good preventive health service delivery and population uptake of these would be likely to respond better to Covid-19 measures. Surprisingly, there is no such linkage. Requiring further study is the relationship with influenza vaccination of citizens over 65 years–higher death rates in more vaccinated countries may be linked to more congregated service provision, or to service fragmentation. Table 3 shows that health system resources and structures had little effect on deaths or December rates, but higher spend per capita on health perversely linked with higher early mortality rates.

Table 2F shows that two informally hypothesised characteristics of the political process–public trust in government and age of prime minister–had no effect. However, total deaths per million were lower in countries where experts surveyed felt that politicians were taking note of scientific advice [13]–but this did not apply to case rates, possibly because scientifically-orientated countries were more rigorous in seeking and analysing cases.

### Assessing robustness and resilience of responses

Responses to a health emergency are dynamic. Table 3 shows the mortality rates reported in Table 1 ranked for the two time points, and for the percentage change in rate. The four initially worst affected countries do not improve their relative position, even though they do control the rate of increase. Many of the best performing countries maintain their advantage, while in between some countries fare much better than others. Seven countries improved over the six-month interval by ten or more rank places—Canada, Denmark, Estonia, Finland, Germany, Ireland and Norway, while four countries dropped by ten or more rank places—Bulgaria, Slovenia, Chile, Mexico, while two—Czechia and Colombia—dropped by twenty or more rank places.

**Table 3:** Changes in death rate ranking over six months.

Country	Deaths per million		Rank		% increase in rate in 6 months
	31/05/2020	31/11/2020	31/05/2020	31/11/2020	
Australia	4.0	35.6	1	4	790
New Zealand	4.6	5.2	2	1	14
Slovakia	5.1	153.7	3	12	2896
Korea S.	5.3	10.3	4	2	95
Japan	7.0	16.4	5	3	133
Latvia	12.7	109.2	6	10	758
Greece	16.8	230.8	7	16	1275
Colombia	17.5	722.6	8	33	4032
Cyprus	19.4	55.9	9	5	188
Bulgaria	20.2	580.7	10	29	2782
Malta	20.4	310.3	11	17	1423
Croatia	25.1	435.1	12	22	1634
Lithuania	25.7	185.9	13	14	623
Poland	28.0	453.2	14	24	1517
Iceland	29.3	76.2	15	8	160
Czechia	29.8	777.6	16	34	2510
Israel	32.8	331.8	17	19	911
Norway	43.5	61.2	18	6	41
Estonia	47.5	89.0	19	9	87
Slovenia	52.0	690.3	20	32	1229
Chile	52.2	806.1	21	35	1446
Turkey	53.5	163.0	22	13	205
Hungary	54.2	499.3	23	25	821
Finland	57.0	72.0	24	7	26
Romania	65.1	589.0	25	30	804
Austria	74.2	353.5	26	20	377
Mexico	75.9	821.7	27	38	983
Denmark	98.6	144.5	28	11	47
Germany	101.5	199.3	29	15	96
Portugal	136.9	441.8	30	23	223
Luxembourg	175.7	512.8	31	26	192
Canada	187.4	321.8	32	18	72
Switzerland	191.3	556.4	33	28	191
United States	313.5	810.1	34	37	158
Ireland	334.2	415.8	35	21	24
Netherlands	347.3	551.7	36	27	59
France	440.8	809.2	37	36	84
Sweden	454.8	661.5	38	31	45
United Kingdom	550.7	862.4	39	39	57
Italy	551.4	919.2	40	40	67
Spain	560.2	963.9	41	41	72
Belgium	807.1	1436.2	42	42	78

Legend: Green: ranked from 1 to 10; yellow: from 11 to 21; orange: from 22 to 32; red: from 33 to 42.

To reflect comparative response the study devised two indices, based on the rankings in deaths rates and case rates shown in Table 2, and these are presented in Table 4. The Robustness Index reflected the initial weathering of the early pandemic and remaining in a good position, and was calculated from the rank positions on the three incidences, with four points allocated to countries in the top (lowest rate) quartile, dropping to one point in the worst quartile, giving a maximum of 12 and a minimum of 3 points per country. The Resilience Index sought to identify the countries which had most effectively protected their situation by containing the initial death rate with a low six-month increase, and was created by adding to the Robustness Index a further point for every five ranking places improvement between the two dates of mortality analysis, and negative points for every five rank positions dropped.

**Table 4:** Robustness and Resilience Index.

	Robustness Index	Resilience Index
Austria	6	7
Belgium	4	4
Bulgaria	3	0
Croatia	3	2
Cyprus	9	8
Czechia	4	-1
Denmark	7	10
Estonia	8	10
Finland	10	13
France	4	4
Germany	7	9
Greece	7	6
Hungary	3	3
Ireland	8	10
Italy	4	4
Latvia	8	8
Lithuania	5	5
Luxembourg	3	4
Malta	6	5
Netherlands	5	6
Poland	4	3
Portugal	3	4
Romania	4	3
Slovakia	6	5
Slovenia	3	1
Spain	4	4
Sweden	4	5
Iceland	10	11
Norway	11	13
Switzerland	3	4
United Kingdom	4	4
Australia	10	10
Canada	8	10
Chile	5	3
Colombia	5	1
Israel	7	7
Japan	11	11
Korea S.	11	11
Mexico	5	3
New Zealand	12	12
Turkey	6	7
United States	3	3

The 45 quantitative measures were then related to the three impact groupings to capture their link to dynamic change in this period–the results are given in Table 5. Table 5A shows a few weak Demographic and Socio-economic influences–higher GDP per capita was positively linked with lower rates of increase in deaths over six months, and tertiary education enrolment with stronger national robustness. Table 5B shows that societal factors had more influence, in particular the Human Development Index and extent of Internet use, while nearly all Societal Values measures correlated with a higher Resilience index. Table 5C shows considerable effect of strong public trust, especially in resilient countries, while Table 5D reconfirms that public health measures in general had minimal impact, other than a weak effect of influenza immunisation of persons over 65 years of age regarding death rate increase.

**Table 5:** Correlations between selected structural measures and dynamism of impact change.

**5a. Demographic and Socio-economic**																																							
			Popn (mill)		Dependent Popn			Urban Pop. %			Urban Pop (mill)			Pop’n. Den’y			Pop’n 65+ %			GDP per capita				Gini Index			IncomShare low 10%			Mat’al Pov’ty				Tert. Educ. Enrol				Tert. Educ. Comp.	
			2019		2019			~2017			~2017			2018			2019			2019				2017						2019				2018				2010	
**Death Rate 6 months % Increase**																																							
EU 27			-0.25		-0.22			-0.36			-0.29			0.06			0.01			-0.53**				-0.11			0.06			0.06				-0.14				-0.30	
EU++ 31			-0.24		-0.19			-0.39*			-0.27			0.07			0.08			-0.55**				-0.08			0.01			0.11				-0.12				-0.32	
OECD 42			-0.13		-0.22			-0.28			-0.14			0.00			-0.19			-0.54**				0.25			-0.11			0.22				-0.21				-0.23	
**Robustness Index**																																							
EU 27			-0.15		0.07			0.07			-0.13			-0.01			-0.15			0.12				-0.11			0.12			0.01				0.44*				0.41*	
EU++ 31			-0.22		0.03			0.18			-0.21			-0.10			-0.30			0.22				-0.27			0.23			0.08				0.42*				0.31	
OECD 42			-0.18		0.07			0.28			-0.16			-0.01			-0.06			0.17				-0.19			0.04			-0.02				0.40*				0.34*	
**Resilience Index**																																							
EU 27			-0.04		0.15			0.19			-0.02			-0.05			-0.10			0.37*				-0.06			0.09			-0.08				0.32				0.41*	
EU++ 31			-0.13		0.09			0.27			-0.11			-0.12			-0.24			0.43*				-0.20			0.19			-0.05				0.33				0.35	
OECD 42			-0.16		0.14			0.29			-0.14			-0.03			0.04			0.39*				-0.27			0.12			-0.18				0.37*				0.35*	
**5b. Societal Values**																																							
		Human Dev. Index				World Happ’s Index			OECD Life Satisf.			Trust news media			Trust Writtn Press			Int’net Users				Civil Society Partpn				Public Service Fragilit			Good/ v good health				Relig’n Imp’tn				Relig’n Wee’ly		
		2017				2020			2017			2020			2019			2017				2017				2017			2017				2018				2018		
**Death Rate 6 months % Increase**																																							
EU 27		-0.56**				-0.50**			-0.34			-0.43			-0.50			-0.59**				-0.45*				0.49**			-0.21				0.15				0.18		
EU++ 31		-0.60***				-0.54**			-0.39			-0.39			-0.37			-0.62**				-0.49**				0.53**			-0.30				0.20				0.22		
OECD 42		-0.62***				-0.41***			-0.29			-0.32						-0.65***				-0.41**				0.56***			-0.16				0.19				0.32		
**Robustness Index**																																							
EU 27		0.31				0.29			0.28			0.35			0.16			0.37				0.37				-0.22			0.34				-0.16				-0.20		
EU++ 31		0.36*				0.32			0.35			0.35			0.19			0.44*				0.43*				-0.27			0.35				-0.15				-0.23		
OECD 42		0.33*				0.21			0.23			0.16						0.41**				0.19				-0.24			0.27				-0.23				-0.24		
**Resilience Index**																																							
EU 27		0.48*				0.47*			0.39			0.52*			0.41**			0.57**				0.53*				-0.43*			0.25				-0.17				-0.21		
EU++ 31		0.52**				0.49**			0.45*			0.51*			0.39*			0.60***				0.57**				-0.46**			0.31				-0.18				-0.24		
OECD 42		0.52***				0.37*			0.34*			0.35*						0.59***				0.38*				-0.45**			0.23				-0.28				-0.31		
**5c Public Trust and Awareness**																			**5d Public Health**																				
	Confid Health Systm			Confidsocial media			Confid Gov’t			Follow politic on TV			Follow politic social media			Followpolitic on radio					Infant Mort’y				Life Expect			Cur’nt smoke			Cerv’l. Screen				MCV1 Imms				Flu vacc’n > 65
	2017			2017			~2017			2017			2017			2017					2020				2020			2012			2016				2017				2019
**Death Rate 6 months % Increase**																			**Death Rate 6 months % Increase**																				
EU 27	-0.66**			0.22			-0.46*			0.16			-0.41			-0.15			EU 27		0.39*				-0.49**			0.31			-0.31				0.06				-0.56**
EU++ 31	-0.69***			0.26			-0.46*			0.23			-0.29			-0.15			EU++ 31		0.42*				-0.53**			0.38*			-0.36*				0.07				-0.56**
OECD 42							-0.52**												OECD 42		0.40**				-0.51***			0.10			-0.34				0.09				-0.42*
**Robustness Index**																			**Robustness Index**																				
EU 27	0.54*			-0.15			0.17			0.18			0.55*			0.56*			EU 27		-0.06				0.03			-0.04			0.08				-0.18				-0.04
EU++ 31	0.54**			-0.15			0.18			0.15			-0.29*			0.47***			EU++ 31		-0.17				0.13			-0.22			0.09				-0.13				-0.06
OECD 42							0.25												OECD 42		-0.21				0.34*			-0.20			0.09				-0.03				0.10
**Resilience Index**																			**Resilience Index**																				
EU 27	0.61**			-0.23			0.41*			0.11			0.61**			0.51*			EU 27		-0.22				0.19			-0.26			0.23				-0.09				0.18
EU++ 31	0.63**			-0.24			0.40*			0.08			-0.29***			0.48**			EU++ 31		-0.30				0.27			-0.38*			0.24				-0.06				0.12
OECD 42							0.45**									0.51*			OECD 42		-0.32*				0.43**			-0.23			0.23				-0.03				0.14
**5e Healthcare System**																			**5f Political System**																				
	Spend per capita			Doctor /1,000			Health Empl/ 1,000			Hosp. beds /1,000			Acute beds /1000			Health R&D $$$							Trust in Government				Corruptn Perceptn					Taken Scienc Advice				Age of PM			
	2017			2018			2018			2019			2017			2018							2018				2018					2020				2020			
**Death Rate 6 months % Increase**																			**Death Rate 6 months % Increase**																				
EU 27	-0.60***			-0.19			-0.51*			-0.30			0.39			-0.27			EU 27				0.20				-0.66***					-0.10				0.20			
EU++ 31	-0.61***			-0.21			-0.53**			-0.31**			0.41			-0.25			EU++ 31				0.16				-0.69***					-0.03				0.16			
OECD 42	-0.54***			-0.21			-0.49**			0.10			0.16			-0.12			OECD 42				-0.39				-0.63***					-0.23				0.02			
**Robustness Index**																			**Robustness Index**																				
EU 27	0.13			0.21			0.14			-0.30			-0.09			-0.04			EU 27				-0.24				0.31					0.68*				-0.24			
EU++ 31	0.17			0.34			0.28			-0.31			-0.20			-0.11			EU++ 31				-0.21				0.32					0.51				-0.21			
OECD 42	0.03			0.04			0.23			0.10			0.16			-0.23			OECD 42				0.11				0.31*					0.47*				-0.14			
**Resilience Index**																			**Resilience Index**																				
EU 27	0.39*			0.22			0.36			-0.30			-0.19			0.04			EU 27				-0.41				0.58**					0.70*				-0.41			
EU++ 31	0.40*			0.36			0.44*			-0.31*			-0.27			-0.06			EU++ 31				-0.35				0.56**					0.59*				-0.35			
OECD 42	0.26			0.17			0.40*			0.10			0.05			-0.17			OECD 42				0.35				0.54**					0.58***				-0.24			

Note: Asterisks report the level of significance of the correlations as follows:

*** p<0.01,

** p<0.05,

* p<0.1.

The only notable effects of healthcare system characteristics (Table 5E) were that spend per capita and total number of health employees linked positively to subsequently capping earlier rates of mortality–possibly through having increased scope for reallocating resources. Table 5F reiterates the positive effect of taking advice, but gives conflicting effects of (pre-pandemic) perceived corruption.

### The political system characteristics

Turning to non-numerical aspects of the political structure, Table 6 maps the gender of the Prime Minister, the voting system, and whether there was a coalition government against countries ranked by degree of suppression of increase in deaths, and by the Resilience Index.

Of the 42 countries, nine (21.4%) had a female premier, but of the six countries which kept their death rate growth under 50% in the six month period four (66.7%) had a female premier; of the best ranking sixteen, six (37.5%) had a female premier, while there was only one female among the lowest 21 countries (4.8%). For the Resilience Index the top two countries both had female leaders (100%), as did five out of the top eleven (45.4%).

**Table 6:** Political characteristics.

Table 6a Percentage increase in Deaths per Million 31 May– 30 November 2020				
	% increase	Female Premier	Electoral System	Coalition
New Zealand	14	F	PROther	C
Ireland	24		PROther	C
Finland	25	F	PRList	C
Norway	41	F	PRList	C
Sweden	43		PRList	C
Denmark	46	F	PRList	
United Kingdom	56		FPTP	
Netherlands	58		PRList	C
Canada	65		FPTP	C
Italy	66		PRList	C
Spain	66		PRList	C
Estonia	74		PRList	C
Belgium	76	F	PRList	C
France	83		Other	C
Korea S.	94		Other	
Germany	95	F	PROther	C
Japan	131		Other	C
United States	149		FPTP	
Switzerland	151	F	PRList	C
Iceland	160	F	PRList	C
Cyprus	166		PRList	C
Luxembourg	192		PRList	C
Turkey	203		PRList	
Portugal	220		PRList	
Austria	377		PRList	
Lithuania	623	F	Other	
Latvia	758		PRList	C
Australia	782		PROther	C
Romania	795		PRList	C
Hungary	817		Other	
Israel	901		PRList	C
Mexico	967		Other	
Slovenia	1229		PRList	
Greece	1275		PRList	
Chile	1362		PRList	
Malta	1422		PROther	
Poland	1512		PRList	
Croatia	1634		PRList	C
Czechia	2492		PRList	C
Bulgaria	2782		PRList	C
Slovakia	2896		PRList	
Colombia	3815		PRList	
**Table 6b** Resilience Index				
	Index	Female Premier	Electoral System	Coalition
Finland	13	F	PRList	C
Norway	13	F	PRList	C
New Zealand	12	F	PROther	C
Iceland	11	F	PRList	C
Japan	11		Other	C
Korea S.	11		Other	
Denmark	10	F	PRList	
Estonia	10		PRList	C
Ireland	10		PROther	C
Australia	10		PROther	C
Canada	10		FPTP	C
Germany	9	F	PROther	C
Cyprus	8		PRList	C
Latvia	8		PRList	C
Austria	7		PRList	
Israel	7		PRList	C
Turkey	7		PRList	
Greece	6		PRList	
Netherlands	6		PRList	C
Lithuania	5	F	Other	
Malta	5		PROther	
Slovakia	5		PRList	
Sweden	5		PRList	C
Belgium	4	F	PRList	C
France	4		Other	C
Italy	4		PRList	C
Luxembourg	4		PRList	C
Portugal	4		PRList	
Spain	4		PRList	C
Switzerland	4	F	PRList	C
United Kingdom	4		FPTP	
Hungary	3		Other	
Poland	3		PRList	
Romania	3		PRList	C
Chile	3		PRList	
Mexico	3		Other	
Croatia	2		PRList	C
United States	2		FPTP	
Slovenia	1		PRList	
Colombia	1		PRList	
Bulgaria	0		PRList	C
Czechia	-1		PRList	C

Turning to electoral systems, the six countries keeping their death rate growth below 50% all had proportional presentation electoral systems, with the top two having customised variants. The top three in the Resilience Index are similar. By contrast, two of the three countries with First Past the Post electoral systems fare particularly badly.

Coalition governments are often seen as weak, but the top five performers in each analysis had coalitions, and twelve of the top fourteen (85.7%). This compares with an overall 24 (57.1%) of the 42 countries.

### Cluster analysis

**Fig 1:**
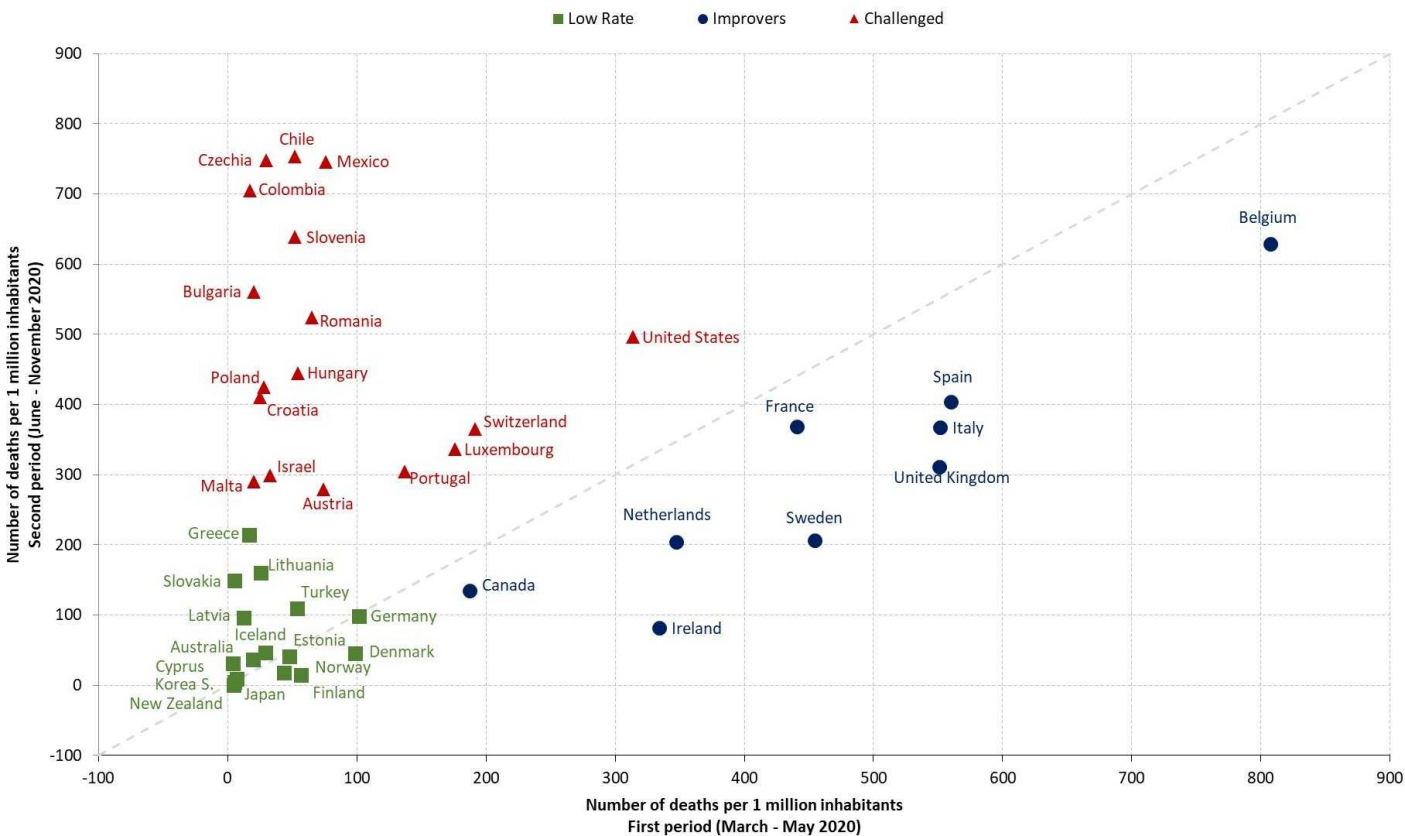
Cluster analysis of 42 countries’ death rates from two time periods.

Next, the study performed a cluster analysis on the death rate data to ascertain if clusters of countries could be identified, and how such clusters relate with the structural variables. This adopted a hierarchical clustering approach that creates a complete data partition applying the Ward method based on the Euclidean distance/proximity matrix [10]. Fig 1 shows the result of grouping countries based on the number of deaths per million inhabitants at the end of May mapped against the increase over the next six months.

In Cluster 1 sixteen Low Rate countries had initial period rates under 100 per million and rates for the subsequent six-months below 200. An Improver cluster of nine countries started with higher initial rates but got control, with their six-month rate lower by between 16% (France) and 76% (Ireland). The third cluster was seventeen Challenged countries, who started above 100 deaths per million and whose second period rates were at least 50% higher than the initial period. These groupings are compatible with and closely match the differently-calculated compilations in the earlier tables.

Table 7 presents the average for each variable for each cluster, and contrasts are highlighted, the limitation being that these cluster averages may be biased by outliers. Most findings from other analyses are confirmed, along with other differences. For Demographic and Socio-economic aspects, the first (Low Rate) cluster is characterized by low population size and density as well as by a high share of citizens having tertiary education, with the opposite for the Challenged cluster, while the Improver cluster has better economic values. Societal Values show the Challenged cluster reports poorer health, more fragile public services, and greatest religious commitment–the opposite to Improvers. Public trust and awareness shows greatest confidence in health systems in Improvers and lowest in Challenged clusters, while for confidence in government, and measures of political awareness, Low Rate countries compare favourably with Improvers.

**Table 7:** Characteristics highlighted by clustering.

	Variable	Average			
		All countries	Cluster 1 Low	Cluster 2 Improver	Cluster 3 Challenged
**Demography and Socio-economic**	Population (mill)	32.5	**24.33**	35.88	38.49*
	Dependent Pop’n. %	53.9	53.94	56.11	52.71
	Urban Pop. %	77.0	78.1	81.58	73.45
	Pop’n. Density sq. Km	167.5	**117.24҂**	187.2	**204.45§**
	Pop’n 65+ %	18.0	18.25	19.22	17.06
	GDP per capita	37653.3	37681	**46737**	**32819**
	Gini Index	33.0	31.8	31.98	34.52
	Income share lowest 10%	2.8	2.79	2.84	2.71
	Living in Poverty %	18.0	**19.96**	**15.19**	18.25
	Tertiary Educat. Enrolm’t	74.2	**85.43**	74.48	**63.48**
	Tertiary Educat. Compl’n	14.8	16.11	**16.16**	**12.86**
**Societal Values**	Human Development Index	0.9	0.89	0.92	0.86
	World Happiness Index	6610.9	6560	6968	6470
	Life Satisfaction OECD	6.6	6.6	6.84	6.53
	Trust in News Media	38.6	40	38.11	37.75
	Trust in Written Press	48.4	50.78	49.5	45.73
	Population using Internet	83.6	86.96	87.61	78.35
	Civil Society Particip’n	0.7	0.75	0.78	0.67
	Public Service Fragility	2.4	2.17	**1.66**	**2.93**
	Good or very good health	68.1	69.73	**71.25**	**65.09**
	Religion Important	9.3	8.31	**6.22**	**12.14**
	Religion Weekly Practice	7.6	6.33	**6.11**	**9.71**
**Public trust and awareness**	Confid in Health System	64.4	70.18	**76.6**	**51.23**
	Confid in social media	19.8	21.35	17.37	20.03
	Confid in Gov’t	34.5	**40.89**	34.79	**27.58**
	Follow politics on TV	51.8	**56.7**	**44.25**	52.51
	Follow politics social media	27.7	**35.67**	**22.68**	24.03
	Follow politics on radio	32.7	**42.13**	**23.19**	30.6
**Public health**	Infant Mortality	4.6	4.34	**3.44**	**5.52**
	Life Expectancy	80.3	80.29	81.99	79.29
	Current smokers %	22.5	22.61	21.52	22.87
	Cerv’l. Screen %	60.4	59.06	64.61	58.94
	MCV1 Imms %	94.6	94.69	93.33	95.19
	Flu vacc’n > 65%	45.7	**37.82**	**59.17**	44.49
**Health system**	Spend $ per capita	3951.6	3749	**5004**	**3586**
	Doctors per 1,000	3.4	3.55	3.49	3.27
	Health Employees per million	50.7	52.72	53.43	46.68
	Hospital beds per 1,000	4.7	**5.32**	**3.48**	4.7
	Acute Hosp beds per 1,000	3.7	**4.16**	**2.85**	3.66
	Health R&D Funding $	1591.0	**568**	1048	**3036ф**
**Political Process**	Trust in Government	43.7	42.69	45.41	49.34
	Corruption Perception	65.5	68.25	73.11	57.94
	Taken Scientific Advice (Study)	55.5	**64.38**	46.63	**45.67**
	Age of PM	51.5	52.79	49.44	55.13
	*Female PM*		**37.5%**	11.1%	**5.9%**
	*Electoral System–PR*		75.0%	**66.7%**	**82.4%**
	*- FPTP*		**0.0%**	**22.2%**	5.9%
	*- Other*		**25.0%**	**11.1%**	11.8%
	*Coalition Gov’t*		62.5%	**88.9%**	**35.3%**

* Skewed by USA (328.2; lowest Malta 0.2).

**҂** Skewed by Australia (3.2).

**§** Skewed by Malta (1514).

Ф Skewed by USA (40660).

Notable contrasts between highest and lowest values per cluster shown in bold.

As with the other analyses, public health activities seem to have little impact. Health system results are varied–Improvers had most spend, and Low Rate countries most beds, while Challenged countries had the highest R&D spend, but these averages were potentially biased by outliers. In political process, Low Rate countries were most likely to take scientific advice, have a female prime minister, their own proportional electoral system, and not to have first-past-the-post elections. Challenged countries took least scientific advice, and were likely to have majority government and a male prime minister. Overall the cluster analysis shows a somewhat cohesive Challenged cluster of countries with signs of resource and democratic leadership disadvantage

### Regression analysis

Having initially considered the countries’ structural variables individually, and recognising the limitations of bilateral correlations as a measurement method in the real world of complex inter-linked health and democratic systems, the study created an econometric model to ascertain how selected variables jointly influenced countries’ fatalities over the full period to November 2020 [14]. Due to the number of observations available, and incomplete data for some, not all the variables could be added to the model. A representative set of ten variables was selected covering all six groupings–as explained in S6 Table.

To estimate the effect of the of the variables selected on our dependent variables, namely the number of fatalities over the entire period to November 2020, the study employed an Ordinary Least Squares (OLS) linear regression estimation model. The choice of employing an OLS model is justified by the fact that that the object of the study is fairly new and the paper rather explorative, and in the absence of particular hypotheses to be tested the OLS represent a parsimonious and useful model.

Since, as already pointed out, most of our explanatory variables show relations that are not linear in parameters, we employed a log-log regression, namely a regression in which both the dependent and the independent variables are converted into natural logs.

The econometric model takes the form of the following equation:

$$Y_{i}=\beta_{0}+\beta_{1}X_{i}+\beta_{2}X_{i}+\cdots +\beta_{k}X_{i}+\mu$$ Eq 1

where y represents the dependent variable, β_0_ the constant term, X represents the independent variables and μ represents the error term.

Substituting the terms with the variables employed in the analysis, the three equations become:

$$\begin{array}{l} Total\;deaths\;per\;million{\;}_{it}\\ \;=\beta_{0}+\beta_{1}\;ln\_Urban\;population\;{\%}_{\text{i}}+\beta_{2}\;ln\_Income\;share\;lowest\;10{\%}_{i}\\ \;+\beta_{3}\;ln\_Tertiary\;education\;enrolment_{i}+\beta_{4}\;ln\_Population\;u\sin g\;Internet_{i}\\ \;+\beta_{5}\;ln\_Civil\;Society\;Participation_{i}+\beta_{6}\;ln\_Confidence\;in\;Govenrment_{i}\\ \;+\beta_{7}\;ln\_Life\;Expectancy_{i}+\beta_{8}\;ln\_MCV1\;Immunization{\%}_{i}\\ \;+\beta_{9}\;ln\_Spend\;\$per\;capita_{i}+\beta_{10}\;ln\_Age\;of\;Prime\;Minister_{i}+\mu \end{array}$$ Eq 2

The results are shown in Table 8, and confirm the strong significance of tertiary education enrolment and the negative effect of per capita health expenditure. Other structural influences confirmed at a lower level are population using the Internet, life expectancy, and confidence in government.

**Table 8:** Multivariate calculation of ten representative variables.

Total deaths per million at to 31 November 2020	log-log
Ln_Urban Population %	1.333
	(1.115)
ln_ Income share lowest 10%	1.031
	(1.035)
ln_ Tertiary Education Enrolment	-1.280***
	(0.431)
ln_ Population using Internet	-7.675**
	(3.327)
ln_ Civil Society Participation	-0.505
	(1.895)
ln_ Confidence in Government	-0.956*
	(0.556)
ln_ Life Expectancy	-19.80*
	(11.52)
ln_ MCV1 Immunization %	-2.372
	(5.406)
ln_ Spend $ per capita	2.362***
	(0.725)
ln_ Age of Prime Minister	0.556
	(1.124)
Observations	40
R-squared	0.465

Robust standard errors in parentheses.

*** p<0.01,

** p<0.05,

* p<0.1.

## Discussion

This study set out to identify the national structural aspects which had positive links to a successful response to the Covid-19 pandemic in its early stages–the period when existing means, resources and methods were perforce the foundation of the response. The intention was to identify what structural characteristics could most usefully be recognised as most influential and thus strengthened to boost health systems and to build forward optimal changes in the ‘post Covid era’. The initial focus was on health system and societal factors, as being the traditional focus of health policy studies–covering service structure characteristics, and characteristics of the population (including values and behaviour) as the focus of service delivery.

Unexpectedly, the analyses showed that no aspects of national or health system structure had major effects on the early pandemic impact, though most successful countries had a population size between two and thirty million (Japan and Germany being the main exceptions). Though there are well reported effects of health inequalities on the impact of Covid-19 within countries [15, 16], surprisingly none of the measures of inequity and poverty showed effects between countries. It was also logically anticipated that greater strength and uptake of structured preventive health programmes would link to greater successful public health response, but this proved not to be the case, suggesting the success of these is a result of a specialist silo approach of each programme rather than salutogenic cultural or lifestyle effects.

Percentage of the population enrolled in tertiary education was one very strong influence; this has also been found in the case of infant immunisation rates in Europe [17]. This was followed by number of users of the Internet, and in Europe following politics on the radio or social media. Tertiary education does more than inculcate technical skills–importantly, it also embeds evidence seeking and analysis, and critical thinking. The Internet, discerningly used, provides important sources of evidence. Wider availability of tertiary education would also suggest greater gender equality, and female education is known to have positive effects on family health behaviour. These measures suggest that an aware informed population, with government committed to applying educational resources, is more likely to respond positively and cooperatively with radical public health measures, fitting co-creation theories [18–20].

High expenditure on advanced therapeutic health services, and indeed on individual preventive health programmes, is potentially fully justifiable in terms of focused intrinsic health gain. However, public health, and its vital role in saving morbidity and mortality, benefits little from therapeutic health spend but is also itself not quantified [21].

### Open democracy

The strongest, and least anticipated, findings come from the political characteristics. The clear linkage between resisting the initial wave of the pandemic or successful recovery from it are strongly associated with proportionate representation, and with the likelihood of having a coalition government and of a female prime minister. These characteristics, and being nations with populations under 30 million, fit with the concept of responsive governments being in touch with their population, and being able to lead in a way which is clear and rationally presented to the population in relation to the evidence. These are not aspects hitherto considered in development of health policy, and indeed lie well outside the traditional arena of health policy. However, they do accord with the view that leadership and governance reflect the effectiveness of coordination structures and strongly correlate with politics, which is claimed as “central in determining how citizens and policy makers recognize and define problems” [22]. Furthermore, Oliver suggests that “governmental priorities are influenced by perceptions of the population affected by a given problem, as well as its severity and cause” [22], while from these results the obverse can also be postulated, that populations respond to the way that problems and solutions are portrayed and openly underpinned with shared evidence and logic.

It can be hypothesised that coalition governments, by being more fragile and needing to keep political partners continuously convinced and thus committed, also have to be persuasive [23], and our findings would support that. Conversely, countries with first past the post elections can result in governments representing only a minority of the population, and single party majority governments can ride out criticism with comparative impunity over several years until the next election. One prior position is that elements of politics—such as government, ideology, power and authority—have important impacts on the distribution of a very wide range of health outcomes [24], and that matches our data. Further, the need to think more holistically and more innovatively, to understand the ordinary citizen, and explain with insight and empathy, have been suggested as skills more often seen in female politicians [25].

This study underscores the findings of a large survey of scientists on whether governments were seeking and following scientific advice [13]. Results are only available for 22 of the 42 study countries, but they correlate strongly with success in Covid control–the most successful countries reported here head the list with 65%-77% perceived recognition of scientific focus (or if not included in that survey have similar evidence of government seeking and being influenced by science, such as Ireland [26, 27]); by contrast the UK with a high mortality rate is low at 24% perceived commitment of scientific input. Overall, the study results also concur with other contemporaneously conducted but differently focussed studies [28–30].

This study completed its analysis just as the WHO-initiated Independent Panel [31] completed its main report [32, 33]. That Panel studied in detail 28 countries, and identified that leadership which failed to base decisions on scientific evidence was a key factor in poor performance in thwarting the pandemic. The work reported here worked from bottom-up data for 42 countries. The commonality of findings is in determining that scientific based open and visible evidence-based leadership was influential in producing the best responses to minimise the impact of the pandemic.

### Theoretical and practical implications

Hitherto consideration of pandemic preparedness has focussed on technical capacity and functions [10], while WHO has also promoted understanding of the impact of a wider range of national and public policies on health in the work on Health in all Policies [34]. The results from this study take this further, by demonstrating on the one hand that wider public policy aspects such as high tertiary education access and uptake have clear health benefits; but secondly that when health crises need rapid realignment of health response and also need change of public behaviour, informative and empathetic democracy styles save lives.

Within a European context, these findings reinforce the relevance and perception of the Tallinn Charter on Health Systems for Health and Wealth [35]. This promoted a range of principles including equity, participation, transparency, and cross-sectoral investment to promote health, and all countries signed up to this programme in 2008. Though not conceived in a pandemic context, nevertheless these values have shown by this study to be more influential in pandemic recovery than health sector specific strengths.

And in a wider context, these findings link in with an emergent debate in countries and primarily in social media about the limitations of some voting systems and government selection methods, particularly the first past the post system which can lead to governments gaining a strong majority based on a minority of voters. Not hitherto considered a determinant of health, representation of (and leadership of) the people may now emerge as important in effective pandemic leadership.

### Limits of the study

This study perforce features some limitations that, even if arguably they do not undermine the core findings, for the sake of completeness need to be presented and discussed.

Firstly, the data available obliged us to take each state as a jurisdictional entity, preventing the possibility of considering the complexity of different forms of federal states, layers of administration such as national and municipal, and the variants of service decentralisation and devolution.

Secondly, despite our efforts to find data for all the countries in our sample, as indicated earlier some values for some indicators were not available. We chose not to seek to approximate missing values from other sources with different definitions, or where a large-scale study omitted individual countries–in other words, we prioritised comparability over approximation-based coverage. Thus some of the variables employed in the correlation analyses presented one or more missing values (i.e. observations that were not available for one or more countries). Some of the correlations, therefore, were calculated based on a partial sample. For these reasons, indeed, the results obtained by the correlations containing missing values are less generalizable and should be referred only for those countries for which the values are present. This applies also for the averages calculations in the cluster analysis. For the sake of clarity, the list of the variables employed and the collected and missing values for each is reported in the Supporting information.

It is important to stress that these considerations do not apply to the econometric analysis for which the variables considered in the model have been selected not least on the basis of the absence (or very low presence) of missing values. Also, the presence of outliers influences the results of the cluster analysis, highlighting the need of further investigations to understand the presence of such extreme values.

Finally, we emphasise that the Demographic and Socio-economic and the Political System measures had very high data completeness, as well as yielding strong findings. The Public Health and Health System measures had scattered small numbers of void cells, due mainly either to a specific preventive programme not being available in some countries, or to use of different reporting characteristics of health resources which could not accurately be recalculated–these data gaps are fairly few and random, and are not likely to have a systemic effect on the overall findings.

## Conclusion

The clear conclusion of this multi-dimensional study is that success in combating the early stages of the Covid-19 pandemic was little influenced by health resources and spend, but was much more beneficially influenced by having an empowering and open style of government. Secondly, governments which already supported their people’s awareness, such as by investing in higher education, and then in the crisis listening to the scientists and explaining to the people from a position of respectful and empathic leadership, fared much better. This fits with the concept of Health in all Policies as the key to the health of populations [34], and with more recent analysis linked to Covid-19 [36]. The style of many politicians and policy makers, in setting a policy dogma on health system style, and promoting higher funding levels, appears to be missing a key point. What seems proven as important and effective is a government focussed on leading the people, explaining problems and resultant policies, with a culture of leadership rather than instruction. This study’s findings also accord with a recent global analysis which identified Canada, Denmark, Finland, Iceland, Ireland, New Zealand and Norway as the countries having the freest forms of democracy [37]—all these countries are shown in our study to have had good early phase Covid outcomes, particularly in effective responses to the initial impact giving rapid corrective action as reported in Table 3

As already stated, this study was limited by the need to find and use impartial comparable data. The study also took each state as a jurisdictional entity, and was not able to consider the complexity of different forms of federal states and variants of service decentralisation and devolution. But at a top level this arguably does not undermine the core findings.

Majority governments, seeming strong but being more impervious to feedback, appear less effective in pandemic management than open and responsive ones. Greater strength is shown by seeking, listening, making evidence-based decisions, and explaining the chosen way forward, and this yields better results. And to enable this leadership style to prevail, a proportionally represented population, empowered in particular by tertiary education opportunity which itself cultivates critical analysis as a social skill, seems important. Replication of this study in other geo-political clusters of like states, such as the Latin American and Caribbean regions, or South East Asia, would also be valuable, as would study of different federal and decentralisation models, and the effects of different electoral systems.

Most recently, Ahmad *et al* have undertaken a large-scale study of 928 respondents in 66 countries, to assess the Political, Economic, Sociological, Technological, Ecological, Legislative and Industry (PESTELI) factors helping and hindering the initial response to the pandemic [38]. Though a very different study, and based on respondents’ views and analyses collected in a framed way rather than on pre-existing structural data, there are commonalities, and little conflict. In particular they found that GDP is not a determinant of a successful response to the pandemic, and that there can be a naïve reliance on technology without understanding of operational systems. But above all, and in line with our initially unexpected findings, they identified that political leadership was often dogmatic and unhelpful, and in some countries science and data were obscured, but in more successful countries leadership which sought and shared facts and views was more appreciated and successful. They also found the move to dogmatically strong leadership could lead to placement of contracts with close associates, and related corruption rather than effectiveness.

Separately, McKee *et al* have undertaken a scoping study suggesting a correlation between right-wing populist leaders and high Covid-19 deaths [39]. In our study and these two more recent and different ones, the political styles in the USA and United Kingdom in early 2020 seem to be linked to globally comparative high mortality rates.

As already cited, studies of specific Covid-related responses are in hand. But to enable better use of the results, more study of styles and successes of political leadership, and analysis of the dynamic of the dialogue through accessing indigenous materials, seems necessary to complement health system and intervention studies, as well as assessing how means of implementing societal acceptance and adoption are as important as technical measures. Open democracy emerges as a previously unrecognised Determinant of Health, which is logical as this political leadership style would seem more likely to recognise and address the already well-established determinants of health [40]. More research is needed and justified on the responsibilities of politicians, the political system, and leadership on determining health in all its forms (from protection against health threats, through physical and societal determinants of health, to adroitness of health systems). In parallel, health research into societal determinants should seek to stimulate and interface with political studies’ assessments of the effect of democratic systems and styles.

These results provide important caveats and qualification to the earlier pre-Covid WHO guidance on preparedness for emergencies [1] by demonstrating that it is not simplistic volume of resources or stridency of government, but informing and empowering the population and the health system, which have key effects and influence outcomes. Furthermore, current studies of different interventions to address the specifics of the Covid-19 pandemic, though important, are only part of the picture because it is the deployment of innovations which matters, and the critical leadership and deployment aspects of these need much further unbiased study, Studies of health systems and interventions need to be complemented by studies into holistic political and governmental leadership on health and society, and need to interact with analyses of democratic forms and agencies in regard to their hitherto under-quantified effect on population health. Government of the people, by empathetic people, for the well-being of the people, based on science, appears proven as the optimum approach, but its structure, processes, enablement and practicalities need further identification and realisation.

## Supporting information

S1 Table Reasons for selection of measures. (PDF)

S2 Table Data values for measures by country. (PDF)

S3 Table Data sources. (PDF)

S4 Table Completeness of coverage for the 42 study countries for each measure. (PDF)

S5 Table Prime ministers and equivalents, February 2020. (PDF)

S6 Table Process for selection of measures for the multivariate analysis. (PDF)
