# Supplementary material for: When Covid-19 first struck: Analysis of the influence of structural characteristics of countries - technocracy is strengthened by open democracy
Source: PLoS One. 2021 Oct 4;16(10):e0257757. doi: 10.1371/journal.pone.0257757 (PMC8489721; doi:10.1371/journal.pone.0257757)
Supplement: S1 Table — (PDF) [file pone.0257757.s001.pdf]

When Covid-19 first struck: analysis of the influence of structural characteristics of countries - technocracy is strengthened by open democracy

Supporting Information S1 Table

Reasons for Selection of Measures

***Note** – Supporting Information S3 Table gives the formal definition, date of original data collection or assembly, and data source used for each item.*

**Demographic and Socio-economic**	
Population (million)	To ascertain whether population size influenced countries' outcome in terms of fatalities or confirmed cases. Larger countries could be hypothesised to sustain greater specialist expertise, including academic and professional experts. Conversely, larger countries might have a greater spread of problems and less nimble response pattern.
Dependent Population. %	Dependent population groups (i.e. age dependency by % of working population) are least able to contribute to the pandemic response. Regardless of Covid-19, they are a burden on the active population.
Urban Population. %	Urban populations live and work in close proximity, therefore may have greater risk of infection. Commuting, and child care where there is no local extended family, may add to this. Conversely, they may have better access to services locally.
Urban Population (mill)	Linking earlier items – it may be the critical mass of people living in urban situations which increases the pandemic risk.
Population Density per sq. km	Hypothesised in several informal commentary sources as being a critical factor, due to close proximity of living.
Population over 65 %	Covid-19 is known to affect older people more seriously, so a higher proportion of population aged over 65 years may pose a greater challenge for pandemic response actions. This population group may be harder to reach by modern media, and may need other services whose delivery may be challenged by Covid-19.
GDP per capita	A country's affluence expressed as Gross Domestic Product (GDP) can be hypothesised as a good measure of the resources it can mobilise to address the pandemic. Living standards may also be higher in richer countries.
Gini Index	A well accepted measure of income distribution, a lower score indicating a more equal distribution, and thus a smaller difference in citizens' income.
Income share lowest 10%	Low income adversely affects health, and in Covid-19 terms a reduced ability to implement distancing and self-isolation. This measure assesses what proportion of national income and economic activity falls to those with the lowest incomes.
Living in Material Poverty %	Material poverty – lacking essential facilities and assets in daily living – may correlate with reduced scope to live with

	social distancing and self-isolation, and may also be a pressure to take less pandemic-safe employment.
Tertiary Education Enrolment	A better-informed population may understand pandemic controls better, and argue in their favour. Persons enrolling in tertiary education can be considered a measure of public investment in education, and of societal commitment to further learning. It will largely involve younger adults.
Tertiary Educational Completion	Based on the preceding hypothesis, this measure looks at the total populations' highest educational achievement. Proportion of the population who have a tertiary education qualification.
**<u>Societal Values</u>**	
Human Development Index	Hypothesising that a more developed society may be more responsive and resilient in the face of the Covid-19 pandemic. Promoted by the United Nations Development Programme, the Human Development Index is a summary measure of average achievement in key dimensions of human development: a long and healthy life, being knowledgeable and have a decent standard of living.
World Happiness Index	Attitude to life may influence response to the pandemic and to related public health measures. The World Happiness Index aims to measure life satisfaction to track the quality of lives as they are being lived in more than 150 countries
Life Satisfaction OECD	Included for similar reasons as the World Happiness Index. OECD self-reported Life Satisfaction measures how people evaluate their life as a whole rather than their current feelings. Respondents are asked to rate their general satisfaction with life on a scale from 0 to 10.
Trust in News Media	Populations with greater trust in news media are likely to be better informed about the pandemic, and about the nature of, and reasons for, public health measures and other policy responses. Media are usually live, and often interactive.
Trust in Written Press	Included for the same reasons as Trust in News Media, this recognises that the written press has a different reference value.
Population using Internet	The internet can be a source of both good and misleading information. Overall use is not the same as discriminating fact-finding. This measure is included to ascertain if it has a positive, negative, or no effect on response to the pandemic.
Civil Society Participation	A population which is interactive is hypothetically more likely to be mutually supportive and to have cohesion for the common good.
Public Services Fragility	More robust services may produce better results, both in their direct actions and by instilling greater trust and compliance. This measure of public service fragility is created by the Fragile States Index and used by the World Bank, to assess perception as to how robust, accessible and equitable are key public services.

Good or very good health	People with good health may be more committed to protecting this status. Conversely, populations with less perceived health may be more committed to acting against any further deterioration. This is a survey measure of citizens' subjective perception of their own health.
Religion Important	Most major religions promote societal duty, and not doing harm to others – indeed, they promote good works. Countries with a higher level of recognition of the importance of religion might be expected to promote and sustain societal public health measures.
Religion Weekly Practice	Persons practicing religious celebration regularly might be expected to be conscientious with regard to personal protection and protection of others. They will also be more exposed to exhortations from respected leaders for responsible behaviour.
**<u>Public Trust and Awareness</u>**	
Confidence in Health System	The population is more likely to comply with a health system in which it trusts.
Confidence in social media	Citizens who trust social media are more likely to make informed use of material found on line.
Confidence in Government	Citizens who express trust in Government are more likely to follow Government advice.
Follow politics on TV	It can be hypothesised that citizens who most actively follow politics on television will follow government advice.
Follow politics social media	Included for similar reasons to the above, but based on those using social media to follow politics – the coverage and the user selection may be more balanced – or more biased.
Follow politics on radio	Included for similar reasons to the above, with radio through its ubiquity and less demanding nature (can be used while driving or working) potentially offering more opportunity to listen to breaking news and to ongoing commentary.
**<u>Public Health</u>**	
Infant Mortality	Infant mortality can be postulated as a measure of overall health system preventive health programmes, citizen's commitment to healthy lifestyle, and effectiveness of preventive health service uptake.
Life Expectancy	Life expectancy can be hypothesised as a measure of the outcome of health system performance across therapeutic and preventive programmes, interlinked with personal salutogenic behaviour of the population.
Current smokers %	Hypothesising that a health-conscious population may address Covid-19 controls more assiduously, this item is taken as an available measure of citizen health-related behaviour, and of the degree of preventive health programmes.
Cervical Screening %	A second measure of the hypothesised commitment to a preventive health programme and the interaction of the

	population in availing of the service is uptake by women of cervical screening.
MCV1 Immunisation %	Measles-containing vaccine (MCV) child immunisation is hypothesised to be a measure of population uptake of a preventive service, modified by resistance or low motivation – as infants are adult-dependent this is a potential sensitive measure of societal commitment to protect the vulnerable, and of service persistence to encourage uptake.
Flu vaccination > 65 %	This may measure commitment of preventive health services to the health of the elderly, and of society in prioritising this objective. Annual immunisation against influenza of persons over 65 years is an established preventive health programme, but undertaken with different degrees of positivism and investment in different countries; the elderly are prime victims of Covid-19.
**<u>Healthcare System</u>**	
Spend $ per capita	The per capita (current US dollars, purchasing power adjusted) expenditure on health indicates the resources available for healthcare activities. It might be expected to indicate the strength of response to Covid-19.
Doctors per 1,000	Being a prime health expertise, the number of doctors related to population might be expected to measure the strength of response to any health crisis.
Health Employees 1,000	Covering all health and care employees, this could be expected to indicate the number of health-and-care staff potentially available to deal with pandemic issues.
Hospital beds per 1,000	Included to assess the impact of the level of this prime physical resource for treating the most sick patients, though the proportion of beds fully committed to long term care such as enduring psychiatric problems or chronic disease will vary country by country.
Acute Hosp beds per 1,000	Acute hospital beds might be considered to relate more closely to the facilities needed for very sick Covid-19 patients, though there are limitations to data definitions and to data availability.
Health R&D Funding $	Countries with highest spending on health research and development might be expected to have a greater cohort of expertise and equipment, some of which may be available for redeployment quickly, as well as showing commitment to a more evidence-based culture.
**<u>Political Process</u>**	
Trust in Government	The population might be expected to comply more closely with a government it trusts. This measure is assessed by OECD on the degree to which the public trust their government.
Corruption Perception	Populations may be less compliant with a government they feel is influenced by corruption. This measure is reported by

	the World Bank as to the perception of corruption in government.
Taken Scientific Advice (Study)	A government which takes note of scientific advice regarding Covid-19 might be expected to have better outcomes. This measure is based on a survey by the publication Frontiers in Public Health of scientific experts in each country as to whether government was seen as adopting scientific expert advice.
Age of PM	Older Prime Ministers could be hypothesised to have greater life experience and practical wisdom; or alternatively to be more likely to be enveloped in an isolated ruling class bubble. Younger leaders could be hypothesised to lack experience, or alternatively to be closer to normal citizens' daily lives, use newer information sources, and to understand practicalities and wider effects.
PM Gender	To test the populist assumption in some news and other media outlets which have suggested female leaders may have more intuitive assessment, and greater powers of convincing explanation which instil greater following of challenging policies.
Voting System	There are many different national voting systems; some commentators argue that forms of proportional representation mean that the spread of public opinion and preferences represented ensures that more people feel represented and will therefore have greater practical faith in government decisions.
Coalition Government	It can be argued that one party majority government can in the short term operate against public opinion with impunity: this may enable difficult decisions or conversely unpopular or irresponsible ones. Coalition government can be argued as being weak by having to operate on consensus; or conversely that the need to explain the basis of every decision, not least to avoid parties leaving the coalition at any time, makes for openness and logical argument.
