# Supplementary material for: When Covid-19 first struck: Analysis of the influence of structural characteristics of countries - technocracy is strengthened by open democracy
Source: PLoS One. 2021 Oct 4;16(10):e0257757. doi: 10.1371/journal.pone.0257757 (PMC8489721; doi:10.1371/journal.pone.0257757)
Supplement: S2 Table — (PDF) [file pone.0257757.s002.pdf]

When Covid-19 first struck – the influence of countries' socio-demographic, societal, and system factors on initial outcomes

Supporting Information S2 Table

Data Values for Measures by Country

Note *– Supporting Information S1 Table gives the reason for selection of each data item.*

Note *– Supporting Information S3 Table gives the formal definition, date of original data collection or assembly, and data source used for each item.*

Table 1. Structural Measure Values

1a. Demographic and Socio-economic												
	Popn (mill)	Dependent Popn %	Urban Pop. %	Urban Pop (mill)	Pop'n. Den'y	Pop'n 65+ %	GDP per capita	Gini Index	Incom Share low 10%	Mat'al Pov'ty	Tert. Educ.Enrol	Tert. Educ.Comp.
	2019	2019	~2017	~2017	2018	2019	2019	2017		2019	2018	2010
Austria	8.9	50	58.1	5.2	107.1	19	50,277	29.7	3	3.0	86.7	9.4
Belgium	11.5	56	98.0	11.3	377.4	19	46,117	27.4	3.3	15.1	78.9	17.7
Bulgaria	7.0	56	74.7	5.2	64.7	21	9,738	40.4	1.9	23.4	71.5	13.1
Croatia	4.1	55	56.7	2.3	73.0	21	14853	30.4	3.4	19.5	67.7	11.0
Cyprus	1.2	44	66.8	0.8	128.7	14	27,858	31.4	2.7		81.3	20.8
Czechia	10.7	55	73.7	7.9	137.7	20	23,102	24.9	4.2	9.7	63.8	7.6
Denmark	5.8	57	87.8	5.1	138.0	20	59,822	28.7	3.7	13.4	81.2	15.0
Estonia	1.3	57	68.7	0.9	30.4	20	23,660	30.4	3	21.1	70.4	18.9
Finland	5.5	62	85.3	4.7	18.1	22	48,686	27.4	3.8		90.3	12.4
France	67.1	62	80.2	53.8	122.3	20	40,494	31.6	3.2	14.2	67.6	10.6
Germany	83.1	55	77.2	64.2	237.3	22	46,259	31.9	2.9	16.7	70.3	13.1
Greece	10.7	56	78.7	8.4	83.3	22	19,583	34.4	2.4	36.0	142.9	22.5
Hungary	9.8	52	71.1	7.0	108.0	20	16,476	30.6	3	14.9	50.3	15.4
Ireland	4.9	55	63.0	3.1	70.7	14	78,661	32.8	3.1	8.2	77.3	26.8
Italy	60.3	57	70.1	42.3	205.4	23	33,190	35.9	1.9	29.9	64.3	6.8
Latvia	1.9	58	68.1	1.3	31.0	20	17,836	35.6	2.3	25.5	93.0	12.4
Lithuania	2.8	55	67.5	1.9	44.7	20	19,456	37.3	2.1	22.2	73.7	15.6
Luxembrg	0.6	43	90.7	0.5	250.2	14	114,705	34.9	2.4		18.6	18.5
Malta	0.5	54	94.6	0.5	1514.5	21	29,416	29.2	3.4	16.3	59.3	10.5
Netherlnd	17.3	55	91.1	15.8	511.5	20	52,448	28.5	3.5	8.8	87.1	15.6
Poland	38.0	50	60.1	22.8	124.0	18	15,595	29.7	3.2	17.6	68.6	11.4
Portugal	10.3	55	64.7	6.7	112.3	22	23,145	33.8	2.7	19.0	65.7	3.3
Romania	19.4	57	53.9	10.5	84.6	19	12,920	36.0	1.6	22.4	51.0	6.6
Slovakia	5.5	46	53.8	3.0	111.3	16	19,329	25.2	2.9	12.3	45.4	8.8
Slovenia	2.1	55	54.3	1.1	103.0	20	25,739	24.2	4.1	13.9	77.1	13.3
Spain	47.1	52	80.1	37.7	93.7	20	29,614	34.7	2.1	21.1	91.1	15.0
Sweden	10.3	61	87.2	9.0	25.0	20	51,610	28.8	3	15.0	72.5	14.9
Iceland	0.4	53	93.8	0.4	33.5	15	66,945	26.8	3.9		73.1	17.9
Norway	5.3	53	81.9	4.3	14.5	17	75,417	27.0	3.3		83.0	12.2
Switzerlnd	8.7	51	73.8	6.4	215.5	19	81,994	32.7	3.1	6.6	61.4	17.9
UK	66.8	57	83.1	55.5	274.7	19	42,300	34.8	2.8	15.0	61.4	15.3
Australia	25.4	54	85.9	21.8	3.2	16	54,907	*34.4*	2.8		107.8	18.5
Canada	37.6	50	81.4	30.6	4.1	18	46,195	33.3	2.7	9.4	70.1	22.7
Chile	19.0	46	87.5	16.6	25.2	12	14,897	44.4	2.3	14.4	90.9	5.9
Colombia	50.3	46	80.5	40.5	44.7	9	6,432	50.4	1.4	28.0	55.3	18.6
Israel	9.1	67	92.3	8.4	410.5	12	43,645	39.0	1.9	22.0	61.5	20.3
Japan	126.3	68	91.5	115.6	347.1	28	40,247	32.9	2.9	16.1	62.4	18.9
Korea S.	25.7	41	81.5	20.9	529.4	15	31,762	31.7	1.9	14.4	95.9	30.0
Mexico	127.6	51	79.9	102.0	64.9	7	9,863	35.4	2.6	46.2	41.5	9.1
N.Zealand	4.9	55	86.5	4.2	18.4	16	42,084		1.8		83.0	15.5
Turkey	83.4	49	74.6	62.2	107.0	9	9,043	41.9	2.2	21.9	113.2	5.3
USA	328.2	53	82.1	269.5	35.7	16	65,118	41.1	1.8	15.1	88.3	26.8

1b. Societal Values											
	Human Dev. Index	World Happ's Index	OECD Life Satisf.	Trust news media	Trust Writtn Press	Int'net Users	Civil Society Partpn	Public Service Fragilit	Good/ v good health	Relig'n Imp'tn	Relig'n Wee'ly
	2017	2020	2017	2020	2019	2018	2018	2017	2017	2018	2018
Austria	0.91	7,294	7.1	40	56	87.71	0.69	1.1	74.4	11.0	7.0
Belgium	0.92	6,864	6.9	45	63	88.66	0.69	2.3		5.0	4.0
Bulgaria	0.81	5,102		33	30	64.78	0.60	4.4	52.1	2.0	1.0
Croatia	0.83	5,505		39	39	72.69	0.57	2.4	53.4	9.0	5.0
Cyprus	0.87	6,159		41	41	84.43	0.65	2.5	75.8		
Czechia	0.89	6,911	6.7	33	49	80.69	0.58	2.6	67.7	1.0	2.0
Denmark	0.93	7,648	7.6	46	59	97.64	0.87	1	73.2	1.0	0.0
Estonia	0.87	6,022	5.7		50	89.36	0.62	2.9	53.5	1.0	1.0
Finland	0.92	7,809	7.6	56	71	88.89	0.79	1	63.6	3.0	0.0
France	0.90	6,664	6.5	23	48	82.04	0.66	1.1	65.9	3.0	4.0
Germany	0.94	7,076	7.0	45	60	89.74	0.81	1.1	62.9	8.0	5.0
Greece	0.87	5,515	5.4	28	29	72.95	0.71	3.8	80.3	22.0	15.0
Hungary	0.84	6,000	5.6	27	44	76.07	0.40	3.2	53.7	9.0	6.0
Ireland	0.94	7,194	7.0	48	51	84.52	0.73	1.4		9.0	12.0
Italy	0.88	6,387	6.0	29	51	74.39	0.70	2.7	65.5	7.0	9.0
Latvia	0.85	5,850	5.9		48	83.58	0.51	2.9		9.0	5.0
Lithuania	0.86	6,215			46	79.72	0.58	3.5	53.6	17.0	11.0
Luxembrg	0.90	7,238	6.9		63	97.06	0.83	2.1			
Malta	0.88	6,773			28	81.40		2			
Netherlnd	0.93	7,449	7.4	52	73	94.71	0.69	1	71.7	6.0	4.0
Poland	0.87	6,186	6.1	45	41	77.54	0.58	2.3	61.3	23.0	29.0
Portugal	0.85	5,911	5.4	56	58	74.66	0.57	2.2	56.5	20.0	16.0
Romania	0.81	6,124		38	57	70.68	0.48	3.8	66.7	20.0	11.0
Slovakia	0.86	6,281	6.2	28	53	80.66	0.58	2.4	71.1	14.0	14.0
Slovenia	0.90	6,363	5.9		38	79.75	0.61	1.5	60.1		
Spain	0.89	6,401	6.3	36	33	86.11	0.62	2.2	78.4	11.0	13.0
Sweden	0.93	7,353	7.3	38	62	92.14	0.71	1.1	73.6	3.0	1.0
Iceland	0.94	7,504	7.5			99.01	0.81	1.2	72.8		
Norway	0.95	7,488	7.6	45		96.49	0.84	1	76.8	5.0	1.0
Switzerlnd	0.94	7,650	7.5	44		89.69	0.79	1	77.2	6.0	6.0
UK	0.92	7,165	6.8	28	15	94.90	0.72	1.6	72.4	3.0	2.0
Australia	0.94	7,223	7.3	38		86.55	0.65	1.5	71.9	1.0	4.0
Canada	0.93	7,232	7.4	44		91.00	0.74	1.5		9.0	6.0
Chile	0.84	6,228	6.5	30		82.33	0.64	4	71.3	21.0	10.0
Colombia	0.75	6,163				62.26	0.51	5.9	74.4	12.0	19.0
Israel	0.90	7,129	7.2			81.58	0.62			3.0	3.0
Japan	0.91	5,871	5.9	37		84.59	0.61	2.2	51.2	8.0	
Korea S.	0.90	5,872	5.9	21		95.90	0.61	1.9	90.4	12.0	9.0
Mexico	0.77	6,465	6.5	39		65.77	0.43	6.8	71.0	16.0	9.0
N.Zealand	0.92	7,300	7.3			90.81	0.60	1.4	71.6		
Turkey	0.79	5,132	5.5	55		71.04	0.34	4.4	77.2	7.0	11.0
USA	0.92	6,940	6.9	29		87.27	0.77	1.6	71.4	17.0	12.0

1c Public Trust and Awareness						
	Confid Health Systm	Confid social media	Confid Gov’t	Follow politic on TV	Follow politic social media	Follow politic on radio
	2017	2017	~2017	2017	2017	2017
Austria	83.65	18.74	39.46	58.97	30.87	49.47
Belgium			31.5			
Bulgaria	25.3	18.01	20.89	73.35	22.11	19.97
Croatia	42.34	17.21	10	47.48	23.19	20.26
Cyprus			34.3			
Czechia	62.35	23.37	18.56	45.61	22.33	28.04
Denmark	75.94	12.49	39.66	50.26	36.58	34.96
Estonia	63.73	20.37	43.45	65.44	39.47	50.17
Finland	83.19	12.36	42.52	45.79	29.22	28.44
France	84.07	14.62	31.63	41.82	15.87	26.61
Germany	65.05	15.46	36.48	70.74	35.48	62.99
Greece			12.9			
Hungary	38.27	23.26	39.21	48.89	23.41	25.75
Ireland			37.6			
Italy	60.76	20.23	24.31	45.84	17.98	14.04
Latvia			21.1			
Lithuania	50.84	48.52	41.62	64.16	40.66	40.21
Luxembrg			67.7			
Malta			52.8			
Netherlnd	74.07	17.42	49.01	42.03	23.16	22.97
Poland	46.34	22.41	24.47	57.84	29.28	39.72
Portugal			34.4			
Romania	46.73	29.11	18.81	57.96	20.91	23.52
Slovakia	55.63	27.22	30.89	55.04	17.76	40.53
Slovenia	45.16	13.77	14.47	38.56	15.33	26.81
Spain	76.46	22.42	22.33	63.44	26.39	28.9
Sweden	81.14	12.91	51.87	39.22	33.04	26.78
Iceland	78.49	22.65	31.41	57.58	52.43	48.78
Norway	88.6	11.75	59.83	44.58	33.72	30.97
Switzerlnd	70.97	14.37	66.9	43.93	28.87	41.86
UK	83.12	16.64	29.61	33.17	19.63	19.82
Australia			30.3			
Canada			36.7			
Chile			36.3			
Colombia			11.9			
Israel						
Japan			39.9			
Korea S.			51.3			
Mexico			17.4			
N.Zealand			50			
Turkey			68.8			
USA			33.4			

1d Public Health						
	Infant Mort’y	Life Expect	Cur’nt smoke	Cerv’l. Screen	MCV1 Imms	Flu vacc’n > 65
	2020	2020	2012	2016	2017	2019
Austria	3.3	81.9	29.3	86.6	94	
Belgium	3.3	81.4	26.1	50.7	96	59.1
Bulgaria	8.1	75	32.3	34.4	93	
Croatia	8.6	76.7	27.9	77.1	93	
Cyprus	7.4	79.3	31.6	64.6	90	
Czechia	2.6	79.3	23.6	53.6	96	21.5
Denmark	3.2	81.2	18.7	64.2	95	52
Estonia	3.7	77.4	23.6	45.9	87	10.2
Finland	2.5	81.3	16.5	69.8	96	49.5
France	3.2	82.2	27.3	57.7	90	51
Germany	3.3	81.1	22.3	80.4	97	34.8
Greece	3.7	81.1	35.5	75.5	97	56.2
Hungary	4.7	76.7	26.6	35.5	99	24.1
Ireland	3.6	81.2	24.3	79.7	92	68.5
Italy	3.2	82.5	22.3	40.9	93	53.1
Latvia	5	75.4	29.1	25.2	98	11.7
Lithuania	3.8	75.5	25.8	50.1	92	14.8
Luxembrg	3.3	82.6	25.3	73.1	99	39.8
Malta	4.6	82.8	22.1	32.3	96	
Netherlnd	3.5	81.7	20.8	60.3	93	62.7
Poland	4.3	78.3	27.2	71.7	93	
Portugal	2.6	81.1	21.6	70.7	99	60.8
Romania	8.7	76	26.4	25.9	90	
Slovakia	4.9	77.8	22.4	46	96	12.5
Slovenia	1.7	81.4	22.2	71.9	93	12.9
Spain	3.2	82	24.7	68.7	97	54.9
Sweden	2.6	82.4	13.6	82.4	97	52.2
Iceland	2.1	83.3	14.4	64.8	93	47.5
Norway	2.5	82.1	16.9	74.4	96	38.2
Switzerlnd	3.5	82.8	19.8	74.5	96	
UK	4.1	81.1	20.1	76.5	92	72
Australia	3.1	82.7	16.3		95	
Canada	4.3	83.4	14.5		90	59
Chile	6.2	79.4	28.6	93		68.3
Colombia	12.3	76.6	11.3		95	
Israel	3.3	83	19.4		98	59.8
Japan	1.9	86	21.3		97	48
Korea S.	3	82.6	24		98	85.1
Mexico	10.7	76.7	9.9		97	
N.Zealand	3.5	82.1	16.8		92	62
Turkey	15.8	75.7	26.5	47.8	96	7
USA	5.3	80.3	15.3		92	68.7

1e Healthcare System

	Spend per capita	Doctor /1,000	Health Empl /1000	Hosp. beds /1,000	Acute beds /1000	Health R&D $$$
	2018	2019	2018	~2019	~2017	2018
Austria	5879.10	5.24	52.47	7.37	5.45	204.74
Belgium	5404.92	3.13	55.36	5.64	5	79.11
Bulgaria	1633.80			7.45		
Croatia	1876.10			5.54		
Cyprus	2624.85			3.40		
Czechia	3040.52	4.04	32.83	6.63	4.11	215.37
Denmark	5794.26	4.19	89.58	2.50	2.54	423.32
Estonia	2427.63	3.48	29.8	4.69	3.45	12.66
Finland	4457.17		77.35	3.28	2.8	66.33
France	5250.45	3.37	58.34	5.98	3.09	1197.48
Germany	6098.20	4.31	71.67	8.00	6.02	2201.66
Greece	2340.17		20.76	4.21	3.6	226.47
Hungary	2115.19	3.38	33.33	7.02	4.27	140.89
Ireland	5896.69	3.34	53.3	2.96	2.78	63.43
Italy	3624.08	4.02	32.57	3.18	2.62	1338.40
Latvia	1895.78	3.30	25.66	5.57	3.3	16.26
Lithuania	2312.96	4.60	33.66	6.56	5.44	13.02
Luxembrg	6047.82	2.98	77.18	4.51	3.77	75.47
Malta	3897.33			4.49		
Netherlnd	5634.53		83.1	3.32	2.78	368.03
Poland	2015.29	2.38	25.82	6.62	4.85	418.86
Portugal	3242.35		38.86	3.39	3.25	132.88
Romania	1576.30		83.92	6.89		23.91
Slovakia	2179.54	3.52	25.05	5.82	4.91	59.22
Slovenia	3158.39	3.18	31.59	4.50	4.2	38.55
Spain	3576.49	4.02	31.21	2.97	2.5	1273.31
Sweden	5828.41	4.27		2.22	2.0	94.01
Iceland	5113.22	3.93	64.07	2.91	2.51	3.22
Norway	6818.35	4.93	106.74	3.60	3.2	556.03
Switzerlnd	8113.94	4.34	81.95	4.53	3.66	13.72
UK	4619.57	2.95	60.67	2.54		3669.52
Australia	5004.87	3.75	68	3.84		940.90
Canada	5199.97	2.80	52.93	2.50	1.97	1348.16
Chile	2305.68		25.09	2.11	1.98	74.74
Colombia	1155.41	2.18		1.71		111.88
Israel	3207.47	3.22	49.89	2.99	2.2	13.90
Japan	4503.68	2.49	64.63	13.05	7.79	1828.06
Korea S.	3213.66	2.39	39.63	12.27	7.14	1932.16
Mexico	1065.95	2.44	9.28	1.38		379.98
N.Zealand	4024.39	3.35	56.73	2.61	2.71	101.73
Turkey	1170.78	1.88	17.53	2.81	2.78	139.77
USA	10623.85	2.61	64.69	2.77	2.49	40660.00

1f Political System

Trust in Government	Corruption Perception	Taken Scienc Advice (Study	Age of PM	*PM Gender Female*	*Voting System*	*Coalit. Gov't = C*
2018	2018	2020	2020	2020	2020	2020
45.3	76	61	39		PR List	
43.7	75	51	44	F	PR List	C
	42		60		PR List	C
	48		50		PR List	C
	59		73		PR List	C
39.3	59		65		PR List	C
55.7	88	68	42	F	PR List	
39.0	73		42		PR List	C
55.0	85		35	F	PR List	C
34.3	72	40	41		Other	C
58.7	80	66	65	F	PR Oth	C
14.3	45	76	51		PR List	
55.7	46		56		Other	
59.7	73		40		PR Oth	C
22.7	52	49	55		PR List	C
26.0	58		55		PR List	C
30.7	59		45	F	Other	
72.7	81		46		PR List	C
	54		43		PR Oth	
63.3	82	65	52		PR List	C
43.7	60	29	51		PR List	
45.7	64	61	58		PR List	
	47		56		PR List	C
34.7	50		44		PR List	
24.3	60		61		PR List	
28.7	58	33	48		PR List	C
51.3	85	55	62		PR List	C
36.5	76		44	F	PR List	C
68.7	84	62	58	F	PR List	C
82.3	85	55	59	F	PR List	C
42.3	80	24	55		FPTP	
45.7	77	59	51		PR Oth	C
62.7	81	56	48		FPTP	C
26.7	67	22	70		PR List	
25.0	36	46	43		PR List	
41.7	61		70		PR List	C
38.3	73	42	65		Other	C
33.0	57	65	69		Other	
66	28		66		Other	
39	87	77	39	F	PR Oth	C
65	41		65		PR List	
73	71		73		FPTP	
