# Supplementary material for: When Covid-19 first struck: Analysis of the influence of structural characteristics of countries - technocracy is strengthened by open democracy
Source: PLoS One. 2021 Oct 4;16(10):e0257757. doi: 10.1371/journal.pone.0257757 (PMC8489721; doi:10.1371/journal.pone.0257757)
Supplement: S3 Table — (PDF) [file pone.0257757.s003.pdf]

When Covid-19 first struck: analysis of the influence of structural characteristics of countries - technocracy is strengthened by open democracy

Supporting Information S3 Table

Data Sources

Note *– Supporting Information S1 Table gives the reason for selection of each data item.*

DEMOGRAPHIC and SOCIO-ECONOMIC

Population (million) 2019

Total national population as obtained by the World Bank from national census and other sources.

https://data.worldbank.org/indicator/SP.POP.TOTL?view=chart

Accessed 25 November 2020

Dependent Population % - 2019

Age Dependency Ratio - World Bank staff estimates based on age distributions of United Nations Population Division's World Population Prospects: 2019 Revision.

https://data.worldbank.org/indicator/SP.POP.DPND?view=chart

Accessed 25 November 2020

Urban Population % - 2017

Percentage of population living in urban settings (as defined by national statistical offices)

https://ourworldindata.org/urbanization

Accessed 19 November 2020

Urban Population (million) - 2019

Computed within the study from the foregoing data.

Population Density per sq. km - 2018

People per square kilometre of land area

https://databank.worldbank.org/reports.aspx?source=2&series=EN.POP.DNST&country=

Accessed 19 November 2020

Population over 65 % - 2019

Persons aged 65 years and over as percentage of total population

https://data.worldbank.org/indicator/SP.POP.65UP.TO.ZS?view=chart

Accessed 25 November 2020

GDP per capita - 2019

Gross Domestic Product (GDP) per capita in US $

https://databank.worldbank.org/reports.aspx?source=2&series=NY.GDP.PCAP.CD&country=

Accessed 18 November 2020

Gini Index - 2017

A recognised measure of inequality of distribution (in this case income), where perfect equality scores 0 and maximum inequality scores 100. This source is computed by the World Bank from data based on primary household survey data obtained from government statistical agencies and World Bank country departments.

https://data.worldbank.org/indicator/SI.POV.GINI?end=2018&start=1967&view=chart

Accessed 18 November 2020

Income share lowest 10%

Percentage share of income held by the low 10% of population in terms of income.

https://datacatalog.worldbank.org/income-share-held-lowest-10-0.

Accessed 19 November 2020

Living in Material Poverty % -2019

Percentage of the population living below the poverty line, based on local surveys and related cut-off basic income points, as compiled for the CIA World Factbook

https://www.indexmundi.com/g/r.aspx?v=69

Accessed 18 November 2020

Tertiary Education Enrolment - 2018

Persons regardless of age enrolled in tertiary education, related to the population in the age-group officially associated with tertiary education. High figures may indicate more older students enrolling, slower completion, or multiple registrations for different courses.

https://ourworldindata.org/tertiary-education

Accessed 19 November 2020

https://databank.worldbank.org/reports.aspx?source=2&series=SE.TER.ENRR&country=

Accessed 9 March 2021

Tertiary Education Completion - 2010

Share of population aged 15 and over who have completed tertiary education

https://ourworldindata.org/tertiary-education

Accessed 19 November 2020

SOCIETAL VALUES

Human Development Index - 2017

The Human Development Index (HDI) is a summary measure of average achievement in key dimensions of human development: a long and healthy life, being knowledgeable and have a decent standard of living.

http://hdr.undp.org/en/indicators/137506#

Accessed 19 November 2020

World Happiness Index - 2020

Compiled from survey measures of life satisfaction to track the quality of lives as they are being lived in more than 150 countries.

https://worldhappiness.report/ed/2020/social-environments-for-world-happiness/

Accessed 20 November 2020

Life Satisfaction OECD - 2017

Assessed by OECD, Life Satisfaction measures how people evaluate their life as a whole rather than their current feelings.

http://www.oecdbetterlifeindex.org/topics/life-satisfaction/

Accessed 20 November 2020

Trust in News Media - 2017

Results of a survey on the level of trust in news media in selected European countries in 2017.

https://www.statista.com/statistics/308468/importance-brand-journalist-creating-trust-news/

Accessed 20 November 2020

Trust in Written Press - 2017

Results of a survey on the level of trust in the written press in the EU 28 countries in 2017.

https://www.statista.com/statistics/454403/europe-trust-in-the-written-press-by-country/

Accessed 20 November 2020

Population using Internet - 2018

https://www.cia.gov/the-world-factbook/field/internet-users/

Accessed 7 January 2021

Civil Society Participation - 2018

A World Bank measurement from an expert survey considering the extent to which the population is engaged in civil society activities, ranging from 0 as low to 1 as high.

https://govdata360.worldbank.org/indicators/h8be2e7d4?country=AUT&indicator=41827&countries=BRA&viz=line_chart&years=1975,2018

Accessed 2 January 2021

Public Services Fragility - 2017

A measure of the fragility of public services, quoted by the World Bank as Public Service Value, with the source incorrectly referred to as the Failed States Index. The source used by the World Bank is the Fragile States Index - https://fragilestatesindex.org/excel/ . Scores range up to 10 as most fragile.

https://govdata360.worldbank.org/indicators/hb1c17281?country=BRA&indicator=28911&viz=line_chart&years=2006,2017

Accessed 2 January 2021

Good or very good health 2017

From the European Values Survey and World Values Survey, the percentage of respondents reporting their own state of health as Very Good or Good.

https://www.atlasofeuropeanvalues.eu/maptool.html

(this link directs to the map, in the searcher "Impression of own health" should be chosen, the results show the Percentage of people that describe their health as Good or Very Good

Accessed 4 January 2021

Good or very good health - WVS (2017-2020)

WVS Database (worldvaluessurvey.org)

the link directs to the online data analysis page, the wave 2017-2020 should be chosen, then the countries should be selected, and then the question (state of health subjective) should be chosen.

Accessed 20 January 2021

Religion Important - 2018

Percentage of the population considering Religion to be important, ascertained by Pew Research Centre's Religion and Public Life Project

https://assets.pewresearch.org/wp-content/uploads/sites/11/2018/06/12094010/Appendix-C.pdf

Accessed 10 December 2020

Religion Weekly Practice - 2018

Percentage of the population reporting taking part in Religious Practice weekly, ascertained by Pew Research Centre's Religion and Public Life Project

https://assets.pewresearch.org/wp-content/uploads/sites/11/2018/06/12094010/Appendix-C.pdf

Accessed 10 December 2020

PUBLIC TRUST and AWARENESS

Confidence in Health System - 2017

Percentage of people that have a great deal or quite a lot of confidence in the health care system

https://www.atlasofeuropeanvalues.eu/maptool.html.

Accessed 4 January 2021

Confidence in social media - 2017

Percentage of people that have a great deal or quite a lot of confidence in social media

https://www.atlasofeuropeanvalues.eu/maptool.html.

Accessed 4 January 2021

Confidence in Government – 2017 (WVS-EVS)

Percentage of people that have a great deal or quite a lot of confidence in Government

https://europeanvaluesstudy.eu/methodology-data-documentation/survey-2017/joint-evs-wvs-2017-2021-dataset/

Accessed 4 January 2021

Follow politics on TV - 2017

Percentage of people who follow politics on television every day or several times a week

https://www.atlasofeuropeanvalues.eu/maptool.html.

Accessed 4 January 2021

Follow politics social media - 2017

Percentage of people who follow politics on social media every day or several times a week

https://www.atlasofeuropeanvalues.eu/maptool.html.

Accessed 4 January 2021

Follow politics on radio - 2017

Percentage of people who follow politics on the radio every day or several times a week

https://www.atlasofeuropeanvalues.eu/maptool.html.

Accessed 4 January 2021

PUBLIC HEALTH

Infant Mortality - 2020

https://www.cia.gov/the-world-factbook/countries/

https://www.cia.gov/the-world-factbook/countries/austria/ (accessed 7.01.2021)

https://www.cia.gov/the-world-factbook/countries/belgium/ (accessed 7.01.2021)

https://www.cia.gov/the-world-factbook/countries/bulgaria/ (accessed 7.01.2021)

https://www.cia.gov/the-world-factbook/countries/croatia/ (accessed 7.01.2021)

https://www.cia.gov/the-world-factbook/countries/cyprus/ (accessed 7.01.2021)

https://www.cia.gov/the-world-factbook/countries/czechia/ (accessed 7.01.2021)

https://www.cia.gov/the-world-factbook/countries/denmark/ (accessed 7.01.2021)

https://www.cia.gov/the-world-factbook/countries/estonia/ (accessed 7.01.2021)

https://www.cia.gov/the-world-factbook/countries/finland/ (accessed 7.01.2021)

https://www.cia.gov/the-world-factbook/countries/france/ (accessed 7.01.2021)

https://www.cia.gov/the-world-factbook/countries/germany/ (accessed 7.01.2021)

https://www.cia.gov/the-world-factbook/countries/greece/ (accessed 7.01.2021)

https://www.cia.gov/the-world-factbook/countries/hungary/ (accessed 7.01.2021)

https://www.cia.gov/the-world-factbook/countries/ireland/ (accessed 7.01.2021)

https://www.cia.gov/the-world-factbook/countries/italy/ (accessed 7.01.2021)

https://www.cia.gov/the-world-factbook/countries/latvia/ (accessed 7.01.2021)

https://www.cia.gov/the-world-factbook/countries/lithuania/ (accessed 7.01.2021)

https://www.cia.gov/the-world-factbook/countries/luxembourg/ (accessed 7.01.2021)

https://www.cia.gov/the-world-factbook/countries/malta/ (accessed 7.01.2021)

https://www.cia.gov/the-world-factbook/countries/netherlands/ (accessed 7.01.2021)

https://www.cia.gov/the-world-factbook/countries/poland/ (accessed 7.01.2021)

https://www.cia.gov/the-world-factbook/countries/portugal/ (accessed 7.01.2021)

https://www.cia.gov/the-world-factbook/countries/romania/ (accessed 7.01.2021)

https://www.cia.gov/the-world-factbook/countries/slovakia/ (accessed 7.01.2021)

https://www.cia.gov/the-world-factbook/countries/slovenia/ (accessed 7.01.2021)

https://www.cia.gov/the-world-factbook/countries/spain/ (accessed 7.01.2021)

https://www.cia.gov/the-world-factbook/countries/sweden/ (accessed 7.01.2021)

https://www.cia.gov/the-world-factbook/countries/iceland/ (accessed 7.01.2021)

https://www.cia.gov/the-world-factbook/countries/norway/ (accessed 7.01.2021)

https://www.cia.gov/the-world-factbook/countries/switzerland/ (accessed 7.01.2021)

https://www.cia.gov/the-world-factbook/countries/united-kingdom/ (accessed 7.01.2021)

https://www.cia.gov/the-world-factbook/countries/australia/ (accessed 7.01.2021)

https://www.cia.gov/the-world-factbook/countries/canada/ (accessed 7.01.2021)

https://www.cia.gov/the-world-factbook/countries/chile/ (accessed 7.01.2021)

https://www.cia.gov/the-world-factbook/countries/colombia/ (accessed 7.01.2021)

https://www.cia.gov/the-world-factbook/countries/israel/ (accessed 7.01.2021)

https://www.cia.gov/the-world-factbook/countries/japan/ (accessed 7.01.2021)

https://www.cia.gov/the-world-factbook/countries/korea-south/ (accessed 7.01.2021)

https://www.cia.gov/the-world-factbook/countries/mexico/ (accessed 7.01.2021)

https://www.cia.gov/the-world-factbook/countries/new-zealand/ (accessed 7.01.2021)
https://www.cia.gov/the-world-factbook/countries/turkey/ (accessed 7.01.2021)

https://www.cia.gov/the-world-factbook/countries/united-states/ (accessed 7.01.2021)

The data was collected by checking each country profile

Accessed 7 January 2021

Life Expectancy -2020

https://www.cia.gov/the-world-factbook/countries/

https://www.cia.gov/the-world-factbook/countries/austria/ (accessed 7.01.2021)

https://www.cia.gov/the-world-factbook/countries/belgium/ (accessed 7.01.2021)

https://www.cia.gov/the-world-factbook/countries/bulgaria/ (accessed 7.01.2021)

https://www.cia.gov/the-world-factbook/countries/croatia/ (accessed 7.01.2021)

https://www.cia.gov/the-world-factbook/countries/cyprus/ (accessed 7.01.2021)

https://www.cia.gov/the-world-factbook/countries/czechia/ (accessed 7.01.2021)

https://www.cia.gov/the-world-factbook/countries/denmark/ (accessed 7.01.2021)

https://www.cia.gov/the-world-factbook/countries/estonia/ (accessed 7.01.2021)

https://www.cia.gov/the-world-factbook/countries/finland/ (accessed 7.01.2021)

https://www.cia.gov/the-world-factbook/countries/france/ (accessed 7.01.2021)

https://www.cia.gov/the-world-factbook/countries/germany/ (accessed 7.01.2021)

https://www.cia.gov/the-world-factbook/countries/greece/ (accessed 7.01.2021)

https://www.cia.gov/the-world-factbook/countries/hungary/ (accessed 7.01.2021)

https://www.cia.gov/the-world-factbook/countries/ireland/ (accessed 7.01.2021)

https://www.cia.gov/the-world-factbook/countries/italy/ (accessed 7.01.2021)

https://www.cia.gov/the-world-factbook/countries/latvia/ (accessed 7.01.2021)

https://www.cia.gov/the-world-factbook/countries/lithuania/ (accessed 7.01.2021)

https://www.cia.gov/the-world-factbook/countries/luxembourg/ (accessed 7.01.2021)

https://www.cia.gov/the-world-factbook/countries/malta/ (accessed 7.01.2021)

https://www.cia.gov/the-world-factbook/countries/netherlands/ (accessed 7.01.2021)

https://www.cia.gov/the-world-factbook/countries/poland/ (accessed 7.01.2021)

https://www.cia.gov/the-world-factbook/countries/portugal/ (accessed 7.01.2021)

https://www.cia.gov/the-world-factbook/countries/romania/ (accessed 7.01.2021)

https://www.cia.gov/the-world-factbook/countries/slovakia/ (accessed 7.01.2021)

https://www.cia.gov/the-world-factbook/countries/slovenia/ (accessed 7.01.2021)

https://www.cia.gov/the-world-factbook/countries/spain/ (accessed 7.01.2021)

https://www.cia.gov/the-world-factbook/countries/sweden/ (accessed 7.01.2021)

https://www.cia.gov/the-world-factbook/countries/iceland/ (accessed 7.01.2021)

https://www.cia.gov/the-world-factbook/countries/norway/ (accessed 7.01.2021)

https://www.cia.gov/the-world-factbook/countries/switzerland/ (accessed 7.01.2021)

https://www.cia.gov/the-world-factbook/countries/united-kingdom/ (accessed 7.01.2021)

https://www.cia.gov/the-world-factbook/countries/australia/ (accessed 7.01.2021)

https://www.cia.gov/the-world-factbook/countries/canada/ (accessed 7.01.2021)

https://www.cia.gov/the-world-factbook/countries/chile/ (accessed 7.01.2021)

https://www.cia.gov/the-world-factbook/countries/colombia/ (accessed 7.01.2021)

https://www.cia.gov/the-world-factbook/countries/israel/ (accessed 7.01.2021)

https://www.cia.gov/the-world-factbook/countries/japan/ (accessed 7.01.2021)

https://www.cia.gov/the-world-factbook/countries/korea-south/ (accessed 7.01.2021)

https://www.cia.gov/the-world-factbook/countries/mexico/ (accessed 7.01.2021)

https://www.cia.gov/the-world-factbook/countries/new-zealand/ (accessed 7.01.2021)

https://www.cia.gov/the-world-factbook/countries/turkey/ (accessed 7.01.2021)

https://www.cia.gov/the-world-factbook/countries/united-states/ (accessed 7.01.2021)

The data was collected by checking each country profile

Accessed 7 January 2021

Additionally it can be also found here: https://photius.com/rankings/2020/population/life_expectancy_at_birth_total_population_2020_0.html (accessed 15 March 2021)

Current smokers % - 2012

Percentage of the population over 15 who smoke daily.

https://ourworldindata.org/smoking

Accessed 8 September 2020

Cervical Screening % - 2016

Percentage of women aged 20-69 years screened within the past 3 years.

https://www.oecd-ilibrary.org/sites/health_glance_eur-2018-41-en/index.html?itemId=/content/component/health_glance_eur-2018-41-en

Accessed 9 December 2020

MCV1 Immunisation % - 2017

WHO/UNICEF Estimates of National Immunization Coverage (WUENIC)

https://www.who.int/teams/immunization-vaccines-and-biologicals/immunization-analysis-and-insights/global-monitoring/data-statistics-and-graphics

Accessed 7 December 2020

Flu vaccination > 65 % - 2019

HEALTHCARE SYSTEM

Spend $ per capita - 2018 or most recent available

Current expenditure on health (all functions), all financing schemes, per capita, current prices, Purchasing Power Parity adjusted, in us dollars.

https://stats.oecd.org/Index.aspx?DataSetCode=SHA

Accessed 7 December 2020

Doctors per 1,000 - 2018 or most recent available

"Practising" or “professionally active” doctors, per 1,000 inhabitants.

https://data.oecd.org/healthres/doctors.htm

Accessed 8 December 2020

Health Employees 1,000 - 2018

Total health and social care employees per million population

https://stats.oecd.org/index.aspx?lang=en

Accessed 8 December 2020

Hospital beds per 1,000 -

https://data.oecd.org/healtheqt/hospital-beds.htm

Accessed 8 December 2020

Acute Hosp beds per 1,000 – 2017 or latest year

Acute hospital beds of all types, per resident population.

https://read.oecd-ilibrary.org/view/?ref=119_119689-ud5comtf84&title=Beyond_Containment:Health_systems_responses_to_COVID-19_in_the_OECD

Accessed 8 December 2020

Health R&D Funding $ -

https://stats.oecd.org/

Accessed 8 December 2020

POLITICAL PROCESS

Trust in Government - 2018

In an OECD study, the share of people who report having confidence in the national government.

https://data.oecd.org/gga/trust-in-government.htm

Accessed 7 December 2020

Corruption Perception - 2018

Reported by the World Bank, calculated to capture perceptions of corruption within the past two years. The data sources are standardized to a scale of 0-100 where 0 equals the highest level of perceived corruption and 100 equals the lowest level of perceived corruption.

https://govdata360.worldbank.org/indicators/h345264a2?country=BRA&indicator=32534&viz=line_chart&years=2015,2018

Accessed 7 December 2020

Taken Scientific Advice (Study) - 2020

Responding to a survey of researchers undertaken by the journal Frontiers in Public Health, the proportion of respondents who believed that political policy makers in their country had sufficiently considered scientific advice, the response ranging from 99 to 3,335 scientists per country..

https://www.frontiersin.org/articles/10.3389/fpubh.2020.621563/full

Accessed 30 November 2020

Ages of PM - 2020

The age of the Prime Minister, or national post equivalent.

Google searches per country

Accessed 10 December 2020

PM Gender – 2020

The gender of the Prime Minister, or national post equivalent.

Google searches per country

Accessed 10 December 2020

Voting System - 2020

The type of voting system in each country.

https://en.wikipedia.org/wiki/List_of_electoral_systems_by_country

Accessed 25 January 2021

Coalition Government - 2020

Identification of all countries with a form of coalition government in place.

https://en.wikipedia.org/wiki/List_of_countries_with_coalition_governments

Accessed 25 January 2021

COVID-19 DATA

Cumulative Covid-19 Deaths per Million, 31 May 2020

https://ourworldindata.org/coronavirus-data-explorer?zoomToSelection=true&time=2020-03-01..latest&country=~EuropeanUnion&region=World&deathsMetric=true&interval=total&perCapita=true&smoothing=0&pickerMetric=location&pickerSort=asc

Accessed 16 November 2020

Cumulative Covid-19 Deaths per Million – 30 November 2020

https://ourworldindata.org/coronavirus-data-explorer?tab=table&zoomToSelection=true&time=2020-03-01..2020-11-30&country=~EuropeanUnion&region=World&deathsMetric=true&interval=total&perCapita=true&smoothing=0&pickerMetric=location&pickerSort=asc

Accessed 14 December 2020

Change to Deaths per Million – Six Months 31 May - 30 November 2020

https://ourworldindata.org/coronavirus-data-explorer?tab=table&zoomToSelection=true&time=2020-05-31..2020-11-30&country=~EuropeanUnion&region=World&deathsMetric=true&interval=total&perCapita=true&smoothing=0&pickerMetric=location&pickerSort=asc

Accessed 14 December 2020

14 Day Case Notification Rate – 4 December 2020

https://www.ecdc.europa.eu/en/publications-data/data-national-14-day-notification-rate-covid-19

Accessed 14 December 2020
