# Supplementary material for: When Covid-19 first struck: Analysis of the influence of structural characteristics of countries - technocracy is strengthened by open democracy
Source: PLoS One. 2021 Oct 4;16(10):e0257757. doi: 10.1371/journal.pone.0257757 (PMC8489721; doi:10.1371/journal.pone.0257757)
Supplement: S4 Table — (PDF) [file pone.0257757.s004.pdf]

When Covid-19 first struck: analysis of the influence of structural characteristics of countries - technocracy is strengthened by open democracy

Supporting Information S4 Table

Completeness of Coverage for the 42 Study Countries for each Measure

Note *– Supporting Information S1 Table gives the reason for selection of each data item.*

Note *– Supporting Information S2 Table gives the data values for each country for each data item.*

Note *– Supporting Information S3 Table gives the formal definition, date of original data collection or assembly, and data source used for each item.*

Variable	Valid observations #	Missing observations #
**Demographic and Socio-Economic**		
Popn (mill)	42	0
Dependent Popn	42	0
Urban Pop. %	42	0
Urban Pop (mill)	42	0
Pop’n. Den’y	42	0
Pop’n 65+ %	42	0
GDP per capita	42	0
Gini Index	39	1
IncomShare low 10%	39	1
Mat’al Pov’ty	35	7
Tert. Educ.Enrol	42	0
Tert. Educ.Comp.	42	0
**Societal Values**		
Human Dev.Index	42	0
World Happ’s Index	42	0
OECD Life Satisf.	35	7
Trust news media	32	10
Trust Writtn Press	28	14
Int’net Users	42	0
Civil Society Partp'n	41	1
Public Service Fragilit	42	0
Good/ v good health	35	7
Relig’n Imp’tn	36	6
Relig’n Wee’ly	35	7
**Public Trust and Awareness**		
Confid Health Systm	23	19
Confidsocial media	23	19
Confid Gov’t	41	1
Follow politic on TV	23	19
Follow politic social media	23	19
Followpolitic on radio	23	19
**Public Health**		
Infant Mort’y	42	0
Life Expect	42	0
Cur’nt smoke	42	0
Cerv’l. Screen	33	9
MCV1 imms	41	1
Flu vacc’n > 65	31	11
**Healthcare System**		
Spend per capita,	42	0
Doctor /1,000	32	10
Health Empl/million	36	6
Hosp. Beds /1,000	42	0
Acute beds /1,000	33	9
Health R&D $$$	38	4

Trust in Government	37	5
Corruption Perception	42	0
Taken Scientific Advice (Study	22	20
Age of PM	42	0
PM Gender	42	0
Voting System	42	0
Coalition Government	42	0
