# Supplementary material for: When Covid-19 first struck: Analysis of the influence of structural characteristics of countries - technocracy is strengthened by open democracy
Source: PLoS One. 2021 Oct 4;16(10):e0257757. doi: 10.1371/journal.pone.0257757 (PMC8489721; doi:10.1371/journal.pone.0257757)
Supplement: S5 Table — (PDF) [file pone.0257757.s005.pdf]

When Covid-19 first struck: analysis of the influence of structural characteristics of countries - technocracy is strengthened by open democracy

Supporting Information S5 Table

Prime Ministers and Equivalents, February 2020

	Name	Age	Female	Change during 2020
Austria	Sebastian Kurz	39		
Belgium	Sophie Wilmès*	44	F	
Bulgaria	Boyko Borisov	60		
Croatia	Andrej Plenković	50		
Cyprus	Nicos Anastasiades	73		
Czechia	Andrej Babiš	65		
Denmark	Mette Frederiksen	42	F	
Estonia	Juri Ratas	42		
Finland	Sanna Marin	35	F	
France	Emmanuel Macron	41		
Germany	Angela Merkel	65	F	
Greece	Kyriakos Mitsotakis	51		
Hungary	Viktor Orbán	56		
Ireland	Leo Varadkar	40		Replaced by Micheál Martin, 59, June
Italy	Giuseppe Conte	55		
Latvia	Arturs Krišjānis Kariņš	55		
Lithuania	Ingrida Šimonytė	45	F	
Luxembourg	Xavier Bettel	46		
Malta	Robert Abela	43		
Netherlands	Mark Rutte	52		
Poland	Mateusz Morawiecki	51		
Portugal	António Costa	58		
Romania	Ludovic Orban	56		
Slovakia	Peter Pellegrini	44		Replaced by Igor Matovič, 46, March
Slovenia	Janez Janša	61		
Spain	Pedro Sánchez	48		
Sweden	Stefan Löfven	62		
Iceland	Katrín Jakobsdóttir	44	F	
Norway	Erna Solberg	58	F	
Switzerland	Simonetta Sommaruga	59	F	
United Kingdom	Boris Johnson	55		
Australia	Scott Morrison	51		
Canada	Justin Trudeau	48		
Chile	Sebastián Piñera	70		
Colombia	Iván Duque Márquez	43		
Israel	Benjamin Netanyahu	70		

Japan	Shinzo Abe	65		Replaced by Yoshihide Suga, 72, Sept.
Korea S.	Chung Sye-kyun	69		
Mexico	Andrés Manuel López Obrador	66		
New Zealand	Jacinda Ardern	39	F	
Turkey	Recep Tayyip Erdoğan	65		
United States	Donald Trump	73		

* Initially in caretaker role
