# Supplementary material for: When Covid-19 first struck: Analysis of the influence of structural characteristics of countries - technocracy is strengthened by open democracy
Source: PLoS One. 2021 Oct 4;16(10):e0257757. doi: 10.1371/journal.pone.0257757 (PMC8489721; doi:10.1371/journal.pone.0257757)
Supplement: S6 Table — (PDF) [file pone.0257757.s006.pdf]

When Covid-19 first struck: analysis of the influence of structural characteristics of countries - technocracy is strengthened by open democracy

Supporting Information S6 Table

Process for Selection of Measures for the Multivariate Analysis

The choice of variables for the multivariate panel analysis has been driven by the joint consideration of three distinct aspects, in order of importance: the number of missing values; the correlation coefficient; and the coefficient of variation. A fourth aspect, namely the guidance offered by the literature has been considered in the choice between similar alternatives.

Indeed, to understand how and to what extent different kinds of structural characteristics influenced countries' response to the pandemic in terms of number of casualties, we modelled the regression's equation by adding one or more variables for each Measurement category identified as per Table 1 (main text).

The first aspect is fundamental and justified by the need of not reducing too much the already small sample (of 42 countries). Therefore, in principle, we considered those variables for which there are no missing values. Secondly, we confronted the variables without missing values by looking together at the correlation coefficients and the coefficients of variation. The former indicates to what extent each variable is related to the number of deaths per million at the end of November, while the latter indicates to what extent each variable varies among the countries considered.

The ratio for looking at such coefficients is that in the absence of a solid theory and proper testable hypotheses, due to the novelty of the issue and to the absence of consolidated literature, and since the aim of the paper is to understand what are the "structural" factors that could have helped countries to better respond to the crisis, it makes sense to look not only at the independent variables that have a stronger relation to the dependent variable, but also at those variables that have a higher variation between countries since these are more likely to be able to explain the different outcomes.

Finally, we recurred to theoretical considerations in some specific cases:

- The share of urban population has been chosen over population size, despite the second shown higher correlation and variation, since the literature on disease transmission highlights how crowded urban spaces have an impact on disease transmission (Alirol et al., 2011; Lee and Wong, 2011; Santos-Vega, Martinez and Pascual, 2016; Liu, 2020).
- Life expectancy has been selected over infant mortality rate on the one hand because the infant mortality has been criticised as a measure of population health since it would narrow the focus on a small part of the population to the exclusion of the rest (Reidpath, and Allotey, 2003). On the other side, mortality rate has been found a useful to assess health status, socioeconomic development, and quality of life in a specific population (Xu et al., 2014).
- Spend on health per capita has been chosen over hospital beds per 1,000 population, despite the latter showing higher correlation and variation, because we considered it as a more comprehensive indicator of the resources available for the national health systems, since it includes all the investments in infrastructure, equipment and personnel, as well as preventive care; by contrast, total beds are not homogenous between countries, or equally available expediently for acute pandemic redeployment, due to different patterns of hospitalisation for chronic conditions, disability, and long-term psychiatric conditions.
- Finally, age of prime ministers has been selected over the corruption perception index, despite the latter shows higher correlation and variation, because the corruption perception index is highly correlated with

the indicator measuring citizens' confidence in government and that would generate multicollinearity issues in the regression that would bias the estimates.

The variables selected are those highlighted in blue.

	Variable	N_missing	Correlation with Deaths / mil. November	Coefficient of variation Sample
**Socio demographic**	Population (mill)	0	0.25	1.751126
	Dependent Pop'n. %	0	0.02	0.104766
	Urban Pop. %	0	0.06	0.157828
	Pop'n. Density sq. km	0	0.24	1.496873
	Pop'n 65+ %	0	0.02	0.230346
	GDP per capita	0	-0.04	0.625959
	Gini Index	1	-0.08	0.165563
	Income share lowest 10%	0	0.17	0.251771
	Living in Poverty %	7	-0.05	0.465966
	Tertiary Educat. Enrolm't	0	-0.28	0.279183
	Tertiary Educat. Compl'n	0	-0.19	0.406226
**Society**	Human Development Index	0	0.32	0.05565
	World Happiness Index	0	0.27	0.108544
	Life Satisfaction OECD	7	0.14	0.107541
	Trust in News Media	10	-0.05	0.245328
	Trust in Written Press	14		0.282422
	Population using Internet	0	0.23	0.111697
	Civil Society Particip'n	1	0.28	0.185481
	Public Services Fragility	1	-0.25	0.573346
	Good or very good health	7	0.17	0.137905
	Religion Important	6	0.06	0.710589
	Religion Weekly Practice	7	0.08	0.81745
**Public trust and Awareness**	Confid in Health System	19		0.275339
	Confid in social media	19		0.399045
	Confid in Gov't	1	-0.43	0.432799
	Follow politics on TV	19		0.209669
	Follow politics social media	19		0.331374
	Follow politics on radio	19		0.371423
**Public health**	Infant Mortality	0	0.09	0.631273
	Life Expectancy	0	-0.05	0.03426
	Current smokers %	0	0.01	0.256879
	Cerv'l. Screen %	9	-0.02	0.296402
	MCV1 Imms %	0	-0.01	0.030731
	Flu vacc'n > 65 %	11	0.25	0.469478

**Health system**	Spend $ per capita	0	0.07	0.521643
	Doctors per 1,000	10	-0.1	0.239179
	Health Employees per 1,000	6	-0.16	0.467802
	Hospital beds per 1,000	0	0.1	0.540602
	Acute Hosp beds per 1,000	9	-0.21	0.392029
	Health R&D Funding $	4	0.18	4.121888
**Political Process**	Trust in Government	1	-0.04	0.360985
	Corruption Perception	0	-0.17	0.241411
	Taken Scientific Advice (Study)	20	-0.57	0.297504
	Age of PM	0	-0.01	0.196711

Santos-Vega, M., Martinez, P. P., & Pascual, M. (2016). Climate forcing and infectious disease transmission in urban landscapes: integrating demographic and socioeconomic heterogeneity. *Annals of the New York Academy of Sciences*, *1382*(1), 44-55.

Lee, S. S., & Wong, N. S. (2011). The clustering and transmission dynamics of pandemic influenza A (H1N1) 2009 cases in Hong Kong. *Journal of Infection*, *63*(4), 274-280.

Alirol, E., Getaz, L., Stoll, B., Chappuis, F., & Loutan, L. (2011). Urbanisation and infectious diseases in a globalised world. *The Lancet infectious diseases*, *11*(2), 131-141.

Liu, L. (2020). Emerging study on the transmission of the Novel Coronavirus (COVID-19) from urban perspective: Evidence from China. *Cities*, *103*, 102759.

Reidpath, D. D., & Allotey, P. (2003). Infant mortality rate as an indicator of population health. *Journal of Epidemiology & Community Health*, *57*(5), 344-346.

Xu, Y., Zhang, W., Yang, R., Zou, C., & Zhao, Z. (2014). Infant mortality and life expectancy in China. *Medical science monitor: international medical journal of experimental and clinical research*, *20*, 379.
